# The best time to become a parent: The relationship between tenure and fertility among academics

**DOI:** 10.1371/journal.pone.0353232

**Published:** 2026-08-12

**Authors:** Olga Gorodetskaya, Agnese Vitali, Valentina Tocchioni, Alessandra Minello

**Affiliations:** 1 Research Institute for the Evaluation of Public Policies, Bruno Kessler Foundation, Trento, Italy; 2 Department of Sociology and Social Research, University of Trento, Trento, Italy; 3 Department of Statistics, Computer Science, Applications ‘G. Parenti’, University of Florence, Florence, Italy; 4 Department of Statistical Sciences, University of Padova, Padova, Italy; Utrecht University: Universiteit Utrecht, NETHERLANDS, KINGDOM OF THE

## Abstract

The interplay between career progression and fertility remains a central theme in the scholarly debate on academia. Past research has largely examined the influence of parenthood on academic careers, revealing a motherhood penalty for women and mixed results for men. The converse—whether and how career advancements shape fertility decisions—remains underexplored. This contribution pioneers the study of the relationship between academic career progression, specifically tenure attainment, and fertility for academic women and men. Drawing on unique primary data from a large sample of Italian academics, this study employs discrete-time event-history models to analyze transitions to first and second childbirths, and logistic regression models to assess short-term fertility intentions by career stage and gender. Results reveal that job stability facilitates the transition to parenthood: tenure significantly increases the hazard of becoming a parent for men and, when obtained before age 35, for women. Tenure also significantly boosts short-term fertility intentions among childless women. Instead, tenure is unrelated to the transition to a second birth for either women or men. The positive association between tenure and fertility raises important concerns. Gender differences in the upper age limits to fecundity translate into shorter reproductive lifespans for women than for men. In academia, these differences are exacerbated by women’s later age of promotion compared to men. This results in a considerably shorter post-tenure fertility window for women, leading to higher rates of childlessness and lower fertility compared to men. Policies aimed at reducing gender disparities in academia should address these differences in fertility by supporting academic women in transitioning to parenthood.

## Introduction

The intersection between career progression and fertility remains a focal point in ongoing discussions about gendered academic careers. Academic women continue to experience slower career advancements and are less represented in top-level positions than men [1]. Previous research has primarily focused on whether and how fertility influences academic careers [2–6]. Findings highlight the existence of a motherhood penalty: having children reduces women’s career prospects in academia [2–4], while results are mixed for men [2,3,5,6]. The converse—whether and how career advancements shape fertility decisions—remains underexplored. Bringing together the literature on gender disparities in academia and the demographic literature on economic uncertainty and fertility, this contribution explores whether fertility decisions among academics may be constrained or fostered by their career outcomes.

Filling this knowledge gap is timely, as current generations of academics face long periods of job-related uncertainty before attaining tenure and do so in an increasingly global job market that may demand multiple relocations. Precarious academic careers may significantly contribute to delayed parenthood as individuals wait for contractual stability, reduced economic uncertainty, and increased income before having children [7–9]. Indeed, demographic research convincingly shows that economic instability and employment uncertainty are negatively associated with realized and intended fertility in the general population, also among the highly educated [10,11].

The link between tenure and fertility is especially salient for women, who generally spend longer periods in precarious positions than men and have slower academic careers [12,13]. Not surprisingly, academic women are more likely than academic men to experience childlessness—whether by choice or circumstance—and to have fewer children overall [8,14–16]. Also, previous research has highlighted a persistent gap between the desired and actual number of children among academic women [8], and the reasons behind this gap remain largely underexplored. Furthermore, parenthood in academia often constrains women’s careers more than men’s. Previous research shows that having a child significantly reduces women’s chances of promotion [16,17] and scientific productivity [2,18–22], and limits their likelihood of attaining top-level academic positions [20]. These disparities are further reinforced by an academic model historically structured around the male-breadwinner “ideal worker”, which prioritizes uninterrupted career trajectories and assumes limited involvement in family life [23,24]. Despite progressive policies in place, women in academia still encounter double standards, particularly when returning from parental leave, reflecting the persistent work-family conflict embedded in academia’s demanding culture [25–27]. This tension is further intensified by the initial physical demands of motherhood and by unequal domestic responsibilities, often resulting in a substantial “second shift”: academic women disproportionately shoulder unpaid labor [28–30], and academic mothers tend to be more involved in parenting than academic fathers [6].

Moreover, decisions regarding whether and when to have children may be constrained by the demands of a tenure schedule and by the biological limitations of one’s reproductive window [29,31,32]. Again, constraints are stronger for women because their fecundity begins to decline earlier than for men.

In academia, the uncertainty of the early-career stage – at least until tenure is achieved – directly impacts fertility decisions, especially for women. For example, in China, academic women in fixed-term contracts have lower reproductive intentions than those in other contractual arrangements [9]. In Italy, promotion to associate professor significantly increases fertility among women [33]. Waiting for tenure often compels academic women to delay childbearing to later ages and, consequently, lowers the likelihood of parenthood compared to academic men and similarly educated women outside academia [9,34]. These challenges also extend to progression to higher-order births: academic mothers are significantly less likely than women in other high‑status professions to have a second or third child [35].

To date, no previous quantitative study has jointly examined fertility behaviors and intentions among academics, comparing women and men. The existing literature has overwhelmingly focused on women, often treating men as a residual category or excluding them altogether, with only sparse, dated exceptions [8]. However, emerging cohorts of academic fathers are experiencing increasing parenting demands as social norms shift toward greater paternal involvement in childcare [6,36,37]. Despite this, academic men continue to have higher completed fertility than academic women, and fatherhood is not always associated with declines in scholarly output [2,38]. Qualitative studies show that academic fathers encounter fewer obstacles in combining their academic careers with family life: they tend to devote less time to housework and childcare and therefore report fewer concerns about work–family trade-offs [39]. Consistent with an unequal division of domestic labor, men generally experience less stress than women when managing both academic and family responsibilities [40].

Gendered roles in childcare are reinforced by unequal parental leave between women and men. In Italy, the context of this study, employed mothers are entitled to five months of paid maternity leave, while employed fathers are entitled, since 2022, to ten days of paternity leave [41]. By contrast, PhD candidates do not have access to parental leave under the same conditions. Instead, their scholarship is suspended and their doctoral enrolment extended during periods of maternity or paternity leave: childbearing during one’s PhD may hence be economically undesirable. Postdoctoral researchers and other early-career academics may either be covered by standard parental leave provisions or by similar suspension mechanisms, depending on their contract type. This institutional framework, combined with the disproportionate use of parental leave by women in the general population (63% of mothers versus only 8% of fathers across all employment categories [42]), may result in academic mothers spending longer periods away from work compared to fathers, thereby reinforcing cumulative disadvantages in career continuity and research productivity.

In this study, we estimate the association between attaining tenure—i.e., exiting employment precarity—and fertility among academics. Specifically, we test whether tenure is associated with an increase in the probability of (RQ1) transitioning to parenthood, (RQ2) transitioning to a second child, and (RQ3) expressing short-term fertility intentions, among academic women and men. Our contribution is threefold: we take a life-course, demographic perspective to gender gaps in academia and investigate the link between career progression and fertility, rather than the commonly investigated link between fertility and career outcomes; we analyze both women and men, filling a gap in a literature that has primarily focused on academic women; and we investigate multiple stages of the fertility trajectory, yielding a comprehensive picture of reproductive behaviors among academics.

## Materials and methods

### Data collection

Using an online survey instrument (Supporting Information, Questionnaire), we constructed a novel longitudinal dataset documenting the career and fertility histories of 2,262 academics employed at Italian universities during the 2021–2022 academic year. Respondents retrospectively reported the occurrence and timing of all professional milestones (PhD completion, hiring, tenure, promotion) and family-related events (childbirths, partnerships), enabling a life-course analysis of academic and family careers. The sample comprises men and women across all academic ranks—postdoctoral researchers, non-tenured and tenured assistant professors, permanent researchers, associate professors, and full professors—and spans all scientific disciplines.

The survey explicitly excluded PhD holders who had left academia before 2022. We also excluded individuals employed in non-standard academic positions within the Italian university system, including doctoral candidates, researchers with temporary or grant-funded contracts, post-graduate fellows, collaborators on coordinated and continuous contracts, and adjunct professors. These roles fall outside the standard faculty career track and were therefore outside the target population of our study. Data collection took place from November 21, 2021, to March 21, 2022, by which point the sample had achieved satisfactory coverage across key sociodemographic strata. Although quotas were not fixed in advance, we monitored the composition of the sample relative to the official population structure of Italian academics—disaggregated by gender, academic rank, and scientific field—as reported by the Ministry of University and Research (MUR).

The survey was disseminated through national academic associations and Italian universities. Scientific associations were identified through searches conducted in Italian, using keywords referring to the type of organisation (e.g., *“association”*, *“society”*), combined with the names of the 14 scientific-disciplinary areas listed in the MUR open data: “*Mathematics and Computer Science”, “Physics”, “Chemistry”, “Earth Sciences”, “Biology”, “Medicine”, “Agricultural and Veterinary Sciences”, “Civil Engineering and Architecture”, “Industrial and Information Engineering”, “Ancient, Philological-Literary and Historical-Artistic Studies”, “History, Philosophy, Pedagogy and Psychology”, “Law”, “Economic and Statistics”, “Political and Social Sciences”*. Once the official website of a relevant scientific association was identified, we searched for contact details, typically those of the secretary or the president. In total, we identified 218 national scientific academic associations with available contact details, covering all 14 disciplinary areas. An email describing the study and requesting the dissemination of the survey was sent to these contacts. In the absence of a response within one week, a follow-up email was sent after one week, and then again after two weeks. A total of 72 scientific associations distributed the survey.

We then relied on the official list of Italian universities available on the MUR website, which includes 61 public and 20 private institutions. For each university, we consulted the institutional website to identify relevant contact persons, typically the secretary, the Chair of the Equal Opportunities Committee (*Comitato Unico di Garanzia*), or the Vice-Rector for research. Through this procedure, we identified 74 universities with suitable contact information. These institutions were contacted by email and invited to disseminate the survey among faculty members. As with the scientific associations, non-responses were followed up with two reminder emails. In total, 37 universities agreed to distribute the survey among their academic staff.

The email’s subject and the questionnaire interface were titled “*Career and family: the life journey of academics in Italy*.” The invitation emphasized broader themes of career trajectories and life paths, encouraging participation regardless of parental status. The introductory message read: “*The purpose of this survey is to explore the life trajectories of scholars in Italy. By completing the questionnaire,* *you will help to gather data for the research project aimed at exploring the relationship between family formation and career progression*.”

According to publicly available data from the MUR, the total eligible faculty population in 2022 was equal to 71,462 academics. The questionnaire was accessed by 4,392 individuals. Of these, 2,443 respondents completed all mandatory questions and, after data cleaning, 2,262 cases were retained for further analysis; the achieved sample corresponds to 3.2% of the target population, a figure consistent with response rates observed in other web-based surveys of academic populations facing heavy workloads and survey fatigue [43].

A comparison between the target population and the final sample shows that their composition is broadly similar across sex, academic rank, age, and disciplinary area, with only a few deviations exceeding five percentage points. Women are overrepresented in the sample by 13 percentage points (54% vs. 41%), and academics aged 30–39 by 7 percentage points. Permanent researchers are underrepresented by 5 percentage points. In terms of scientific disciplines, there are no significant deviations, except for biology, which is overrepresented by 6 percentage points, and medicine, which is underrepresented by 7 percentage points. A detailed comparison is reported in the Supporting Information (Figs S1-S4 in S1 File).

To correct the deviations between the sample and the target population, we applied direct post-stratification weights to our sample. The population quotas (defined by gender, academic rank, and disciplinary affiliation) were calculated from the official microdata on university personnel released by MUR. Age was not included in the weighting procedure because individual-level age data for the entire population are unavailable. Each respondent was assigned a weight based on their membership in one of 168 strata defined by the cross-classification of gender (male/female), academic rank (6 categories), and disciplinary field (14 categories).

Weights were computed as:

**w**_**g,r,d**_ = **P**_**g,r,d**_ **/ S**_**g,r,d**_

where:

**w**_**g,r,d**_ is the weight for individuals in gender group g, academic rank r, and discipline d,

**P**_**g,r,d**_ is the share of that group in the total population (authors’ elaboration of MUR data),

**S**_**g,r,d**_ is the corresponding share in the analytical sample.

Weights greater than 1 indicate underrepresentation in the sample (e.g., the maximum weight is for male permanent researchers in medicine, w = 11.27). In contrast, weights below 1 indicate overrepresentation (e.g., the minimum weight is for female non-tenured assistant professors in earth sciences, which are the highest overrepresented group, w = 0.11). Some strata could not be represented in our sample because no respondents with those characteristics participated in the survey; consequently, the corresponding cells contain missing values. Of the 168 strata, 4 are empty; thus, they do not substantially affect the population structure. The full weighting matrix is reported in the Supporting Information (Table S8 in S1 File). All descriptive statistics and regression models reported in this article use weights.

### Ethics

Before accessing the survey, participants were presented with an electronic consent form. To indicate their consent to participate in the study, participants were required to check a box labeled “I agree.” Respondents who did not check the “I agree” box were unable to proceed with completing the questionnaire.

The ethics self-assessment procedure in place at the University of Trento concluded that the proposed research required a favorable opinion from the privacy office, while the approval from the ethics committee was not necessary. The research received a favorable opinion from the University of Trento’s privacy office, and the related Project Privacy Sheet and Information on the processing of personal data for scientific research purposes were archived on 14/09/2021 by the Administrative Secretary of the Department of Sociology and Social Research.

### Empirical strategy

For **RQ1**, which addresses the transition to parenthood, we estimated three nested discrete-time event history models. Model 1 measures the association between promotion to tenure (a binary, time-varying variable indicating the transition from a non-tenured to a tenured position) and conception leading to a live birth, adjusting for all covariates. Model 2 adds an interaction between attaining tenure and age in order to test whether the association between attaining tenure and fertility varies throughout the reproductive lifespan across genders. This is a key concern, given that fertility declines with age, and this decline begins earlier for women than men. Model 3 replaces the binary tenure indicator with a time-varying variable indicating promotion to four career stages—non-tenured early-career researcher; non-tenured assistant professor; tenured assistant professor; associate and full professor—to assess whether promotions influence fertility differently across the four academic stages.

**RQ2,** which investigates the transition to a second child, applies the same modeling framework in a single baseline specification (Model 4) centered on tenure attainment. All models are estimated separately for men and women using annual person-year data, with robust standard errors clustered at the individual level; descriptive statistics are reported in the Supporting Information (Tab. S1 in S1 File).

To investigate the association between career progression and parenthood, the risk set is formed by childless individuals at the time of PhD completion (N = 1,603, of which 53% are women), and the time at risk starts in the year of PhD completion. This approach reflects that holding a PhD is a prerequisite for an academic career in Italy. For the transition to second births**,** the risk set includes parents of one child, with time at risk beginning one year after the first childbirth (N = 1,052, of which 56% are women). Those who conceived a first child before completing their PhD and had a second child afterward, enter the risk set for the second-child transition in the year when they completed their PhD.

In both analyses, the event of interest is the first or second conception leading to a live birth. The baseline hazard is modeled using the respondent’s age, grouped into four categories: younger than 35, 35–39, 40–44, and 45–49. Observations are censored if individuals reach the age of 49 without experiencing the event of interest (first or second birth) or at the survey year, whichever comes first. In our sample, only five respondents reported having their first child after age 49 (one woman and four men), and six respondents reported having their second child after 49 (two women and four men).

In Models 1, 2, and 4, the key time-varying explanatory variable is attaining a tenured academic position, which captures respondents’ transitions from non-tenured positions (i.e., PhD holders, postdoctoral researchers, and non-tenured assistant professors) to their first tenured roles (i.e., permanent researchers, tenured assistant professors, associate professors, and full professors). In Model 3, the key time-varying explanatory variable is instead the promotion to each subsequent academic rank.

The retrospective nature of the data allows us to reconstruct the transition from PhD completion to subsequent academic positions. Most respondents move from PhD completion into entry-level academic positions and then, eventually, into tenured positions. In a small number of cases, respondents move directly from PhD completion to a tenured position. In the period between PhD completion and their first academic position (eventually following a period of employment outside academia), respondents are classified as “PhD holders”.

We include controls for the PhD cohort, distinguishing between individuals who completed their PhD before or after 2010. This cutoff corresponds to the implementation of the Gelmini reform in Italy (Law 240/2010), which significantly reshaped academic career trajectories. The reform introduced the National Scientific Qualification by disciplinary field, based on standardized criteria set by the MUR, and made it a prerequisite for appointment as associate or full professor. The reform also created two assistant professorship tracks: a junior non-tenured position and a senior tenure-track position. We further control for field of study, categorized into six broad areas: Natural Sciences; Engineering and Technology; Health Sciences; Agricultural and Veterinary Sciences; Social Sciences; and Humanities.

To address **RQ3**, i.e., to examine the association between holding a tenured academic position and respondents’ intentions to have a child in the next three years, we employ logistic regression. This model controls for parenthood status at the time of the survey and is estimated using cross-sectional data, capturing respondents’ characteristics and intentions at a single point in time (Model 5). The target sample consists of women of reproductive age (up to age 45) and men with partners of reproductive age, totaling 724 respondents (52% of whom are women). The original variable consists of four categories: “Certainly not,” “Probably not,” “Probably yes,” and “Certainly yes”. Given the relatively small sample size of respondents who answered this question, we recoded the variable into a binary indicator reflecting the intention to certainly or probably have versus not have a(nother) child within the next three years, a well-established practice in the fertility literature [e.g., 44]. The main explanatory variable for Model 5 is the tenure-parenthood status, a categorical variable distinguishing between non-tenured childless respondents, non-tenured parents, tenured childless respondents, and tenured parents. The sample size for this analysis is smaller than in previous models; thus, to ensure model parsimony, we exclude the exact number of children and detailed academic ranks among covariates. As in previous models, controls include respondents’ age (here, for parsimony, categorized as 35 and younger, 36–40, and 41 and older), PhD cohort, and field of study. Additional controls include partnership status (living with a partner or single) and macro area of residence (North, Center, South/Islands), aimed at controlling for differential fertility patterns across regions and possible disparities in academic trajectories and opportunities, with Northern regions holding a more dynamic academic job market than Southern regions [45]. These additional controls are absent in models of realized fertility due to the lack of retrospective information on residence and partnership status at the time of childbirth. Detailed descriptions of the samples for Models 1–5 are available in the Supporting Information (Tables S1 and S2 in S1 File).

All figures reporting average marginal effects (Figs 2–5) use 83% confidence intervals to facilitate visual comparison across estimates. When comparing two estimates, non-overlapping 83% confidence intervals correspond approximately to a statistically significant difference at the 5% level [46–48]. Substantive conclusions remain unchanged when using 95% confidence intervals.

## Results

**Fig 1:**
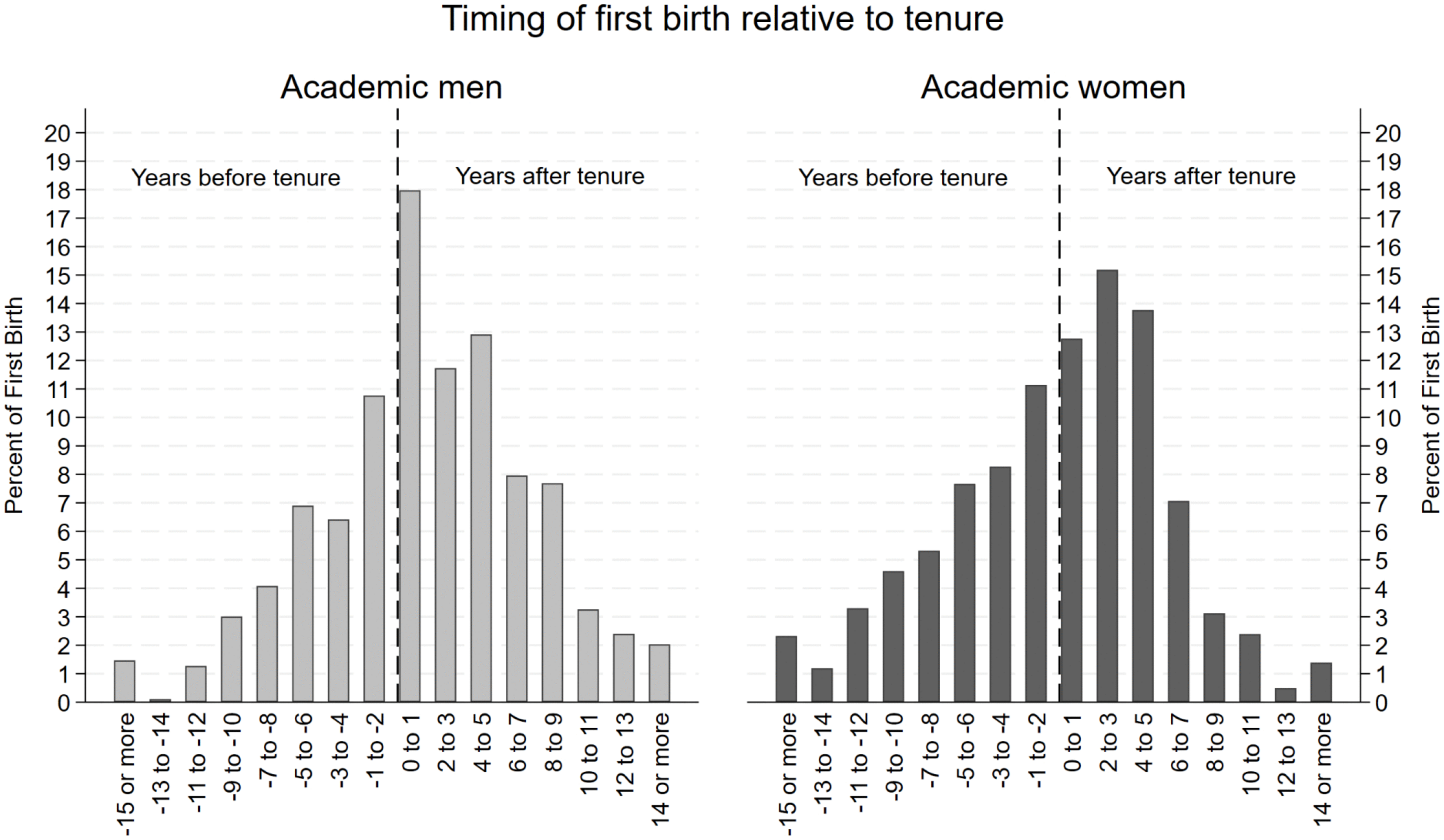
Timing of first birth relative to tenure promotion among academic men (N = 429) and women (N = 452). Note: Only academic parents who had received a tenure promotion by the time of the survey are included.

Academic women transition to parenthood one year earlier than academic men, on average (36 and 37 years old, respectively), a statistically significant difference (Supporting Information, Table S3 in S1 File). The average interval between the first and second child is approximately two years for both men and women, indicating similar birth-spacing patterns among academic mothers and fathers. Fig 1 displays the distribution of first births relative to the year of tenure attainment (marked by the dashed vertical line) for academic men and women. The x-axis represents the number of years before and after tenure (with year 0 indicating the year when tenure is attained), while the y-axis reports the percentage of individuals whose first child was born within each time interval. The figure shows that men tend to experience parenthood around the time of tenure, with a pronounced peak in the year of tenure attainment. By contrast, the timing of parenthood is more heterogeneous among women, who are also more likely than men to become parents before reaching tenure.

**Fig 2:**
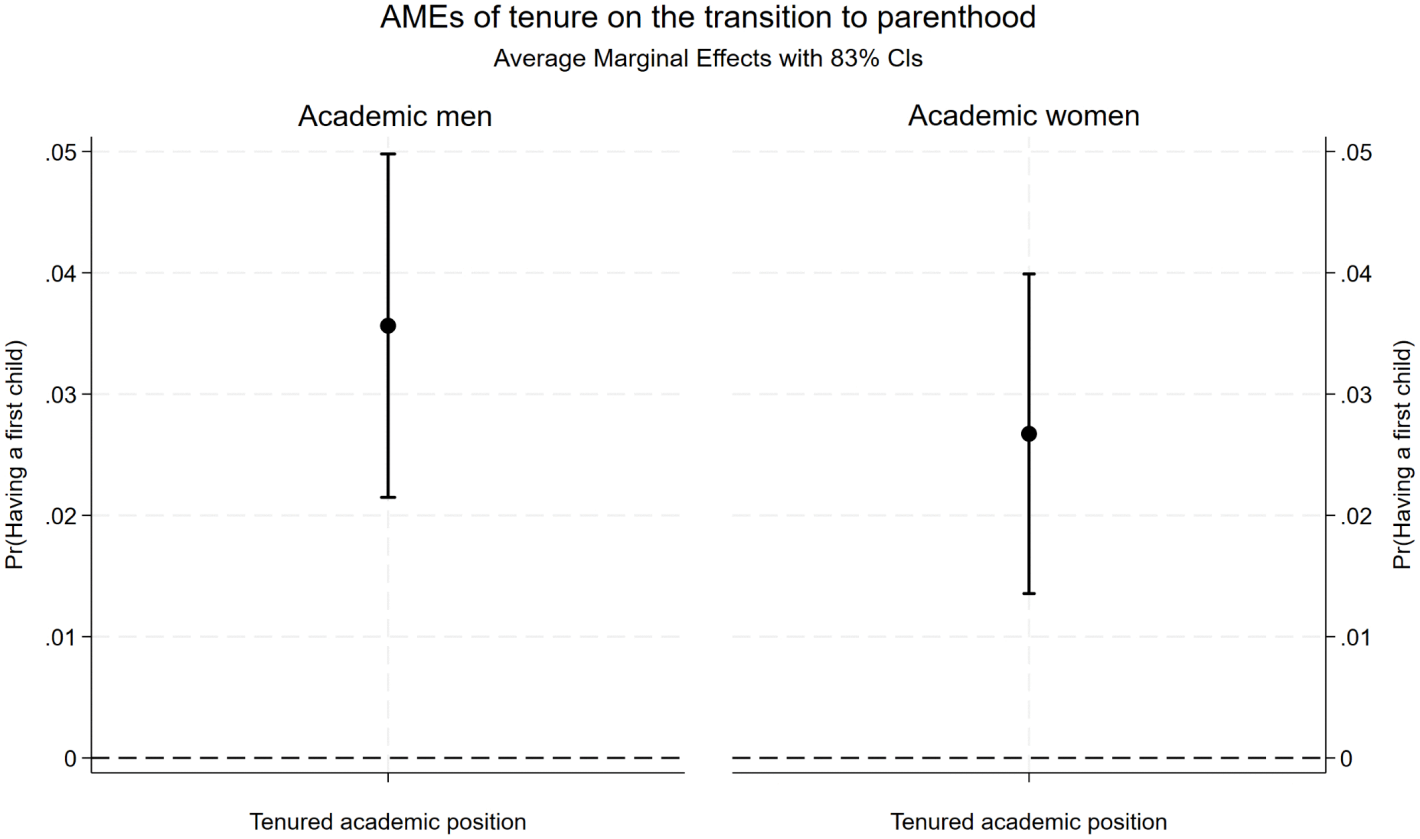
Average marginal effects (AMEs) of tenure versus non-tenure on the probability of becoming a parent among academic men and women: results from Model 1 controlling for age class, field of study, and PhD cohort.

The results of Model 1 (Table 1) show that, after controlling for age, PhD cohort, and field of study, attaining a tenured position significantly increases the hazard of becoming a parent. Among academic men, the hazard of having a first child in the years following promotion increases by 62% compared to non-tenured men (OR = 1.62, p < 0.001), while among academic women it increases by 44% compared to non-tenured women (OR = 1.44, p < 0.01). The average marginal effects (AMEs) of tenure on the probability of having a first child are slightly higher for academic men than women (but the difference is not statistically significant because of overlapping CIs; see Fig 2).

**Table 1:** Discrete-time event history regression model on the transition to parenthood by gender. Results from Model 1.

	Academic Men		Academic Women	
	OR	S.E.	OR	S.E.
*Tenure status (ref. Non-tenured):*				
Tenured	1.62***	(0.219)	1.44**	(0.182)
*Age class (ref. Younger than 35):*				
35–39	1.36*	(0.193)	1.13	(0.151)
40–44	1.11	(0.252)	0.58**	(0.107)
45–49	0.24***	(0.092)	0.09***	(0.045)
*Field of study (ref. Natural sciences):*				
Engineering and Technology	1.06	(0.173)	1.28	(0.234)
Health sciences	1.31	(0.280)	1.03	(0.191)
Agricultural and Veterinary Sciences	0.94	(0.188)	0.88	(0.192)
Social sciences	1.10	(0.197)	1.18	(0.161)
Humanities	1.08	(0.222)	0.64*	(0.118)
*PhD cohort (ref. 2009 and earlier):*				
After 2010	0.99	(0.146)	0.95	(0.132)
Person-years	5,241		5,995	

Note: * p < 0.05, ** p < 0.01, *** p < 0.001

Model 1 also shows that, among academic women, the probability of becoming a mother decreases significantly after age 40, with no difference between women younger than 35 and those aged 35–39. In contrast, for men, the probability of becoming a father peaks between ages 35 and 39 and significantly declines only after age 45.

**Fig 3:**
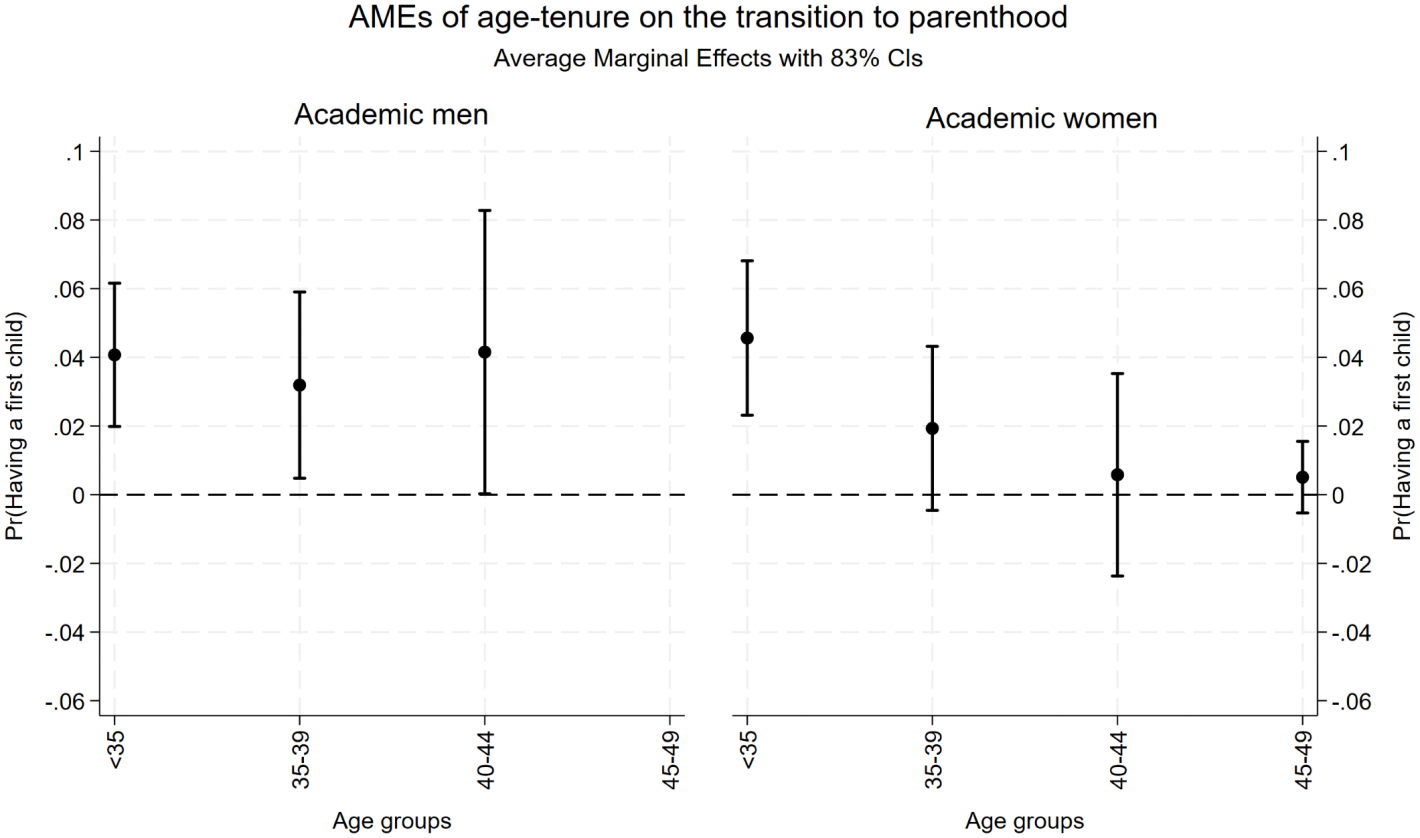
Average marginal effects (AMEs) of age-tenure group on the probability of becoming a parent among academic men and women: results from Model 2, controlling for field of study and PhD cohort. Note: There are too few non-tenured men aged 45–49 who become parents in the sample; hence, the corresponding coefficient cannot be estimated, and this category is omitted.

Results from Model 2 (Fig 3) suggest that academic women who achieve tenure before age 35 have a higher hazard of becoming mothers than women who do not attain tenure by this age. However, as age increases, this difference becomes less pronounced and ultimately statistically insignificant. In contrast, for academic men, the tenure-fertility premium remains positive and statistically significant until the age of 44.

Overall, the transition to a first birth is not significantly associated with PhD cohort nor field of study, except for women in the Humanities, who are significantly less likely to have a first birth than those in the Natural Sciences (Supporting Information, Table S4 in S1 File).

**Fig 4:**
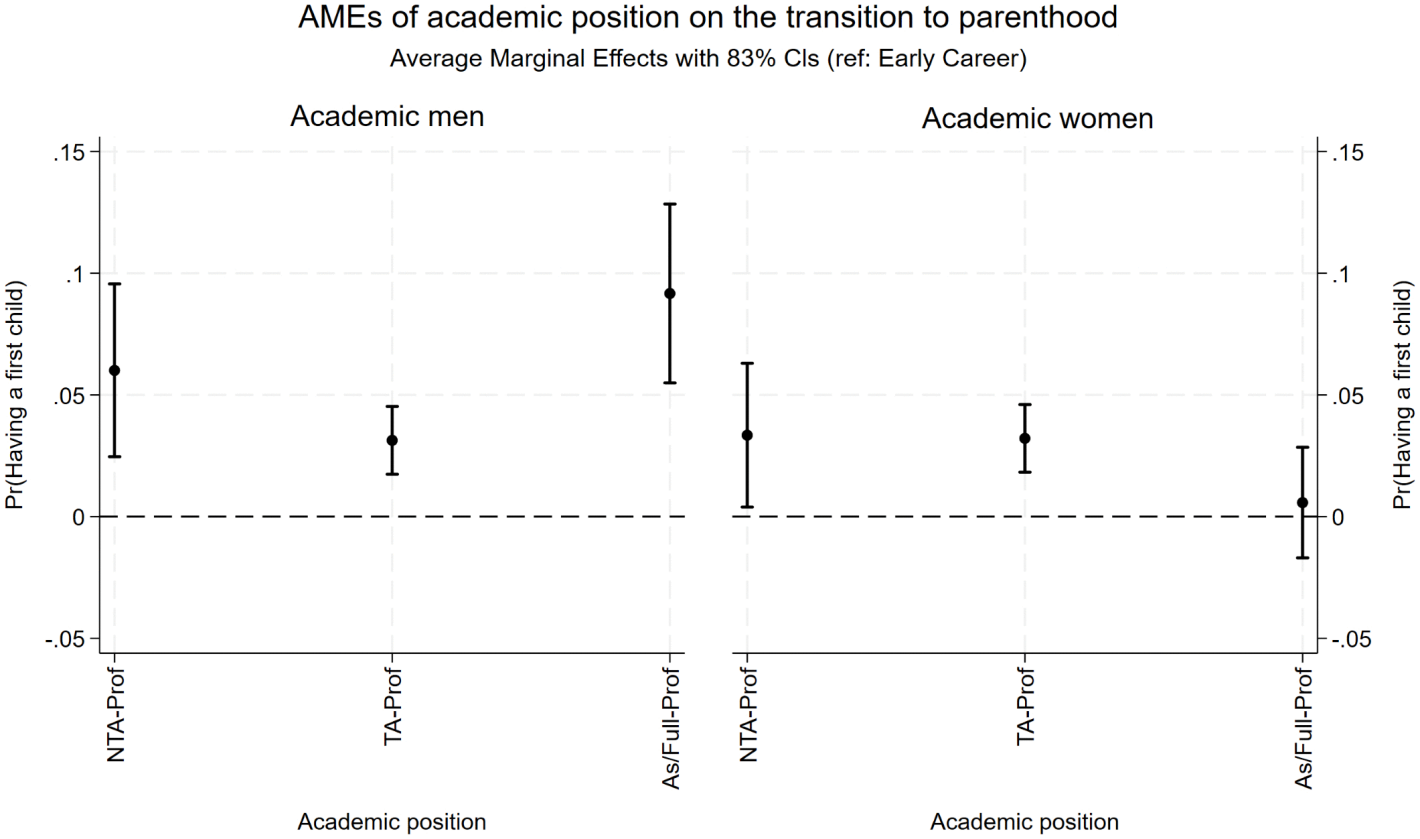
Average marginal effects (AMEs) of academic position on the probability of becoming a parent among academic men and women: results from Model 3 controlling for age class, field of study, and PhD cohort. Note: NTA-Prof = Non-Tenured Assistant Professor; TA-Prof = Tenured Assistant Professor; A/Full-Prof = Associate or Full Professor. Reference category: early-career researchers.

Results of Model 3 (Fig 4) on the impact of attaining a specific academic position on the transition to parenthood (SI, Table S5 in S1 File) show that for men, the highest propensity to become a father is observed among associate and full professors (AME = 0.10). Also, compared with early-career academics (such as PhD graduates seeking employment or postdoctoral researchers), attaining both tenured and non-tenured assistant professorships significantly increases the probability of transitioning to fatherhood (AME = 0.03 and AME = 0.06, respectively). Regarding women, the highest propensity to become a mother is found among tenured assistant professors, i.e., the first stable academic rank (AME = 0.03). Attaining a non-tenured assistant professorship also increases the probability of becoming a mother (AME = 0.03) compared with early-career researchers. However, this difference is not statistically significant at the 95% confidence level. Conversely, the association between attaining full or associate professorships and the transition to parenthood is negligible and statistically insignificant, possibly reflecting the older age at which such career milestones are reached.

Regarding the transition to a second child, Model 4 shows no significant differences between tenured and non-tenured positions for both men and women (SI, Table S6 and Fig S5 in S1 File). The hazard of becoming a second-time parent among tenured academics is comparable to that of non-tenured peers. Overall, the propensity to have a second child declines with age, starting at 40, for both men and women. None of the other factors included in the model, such as PhD cohort or field of study, is statistically significant.

**Fig 5:**
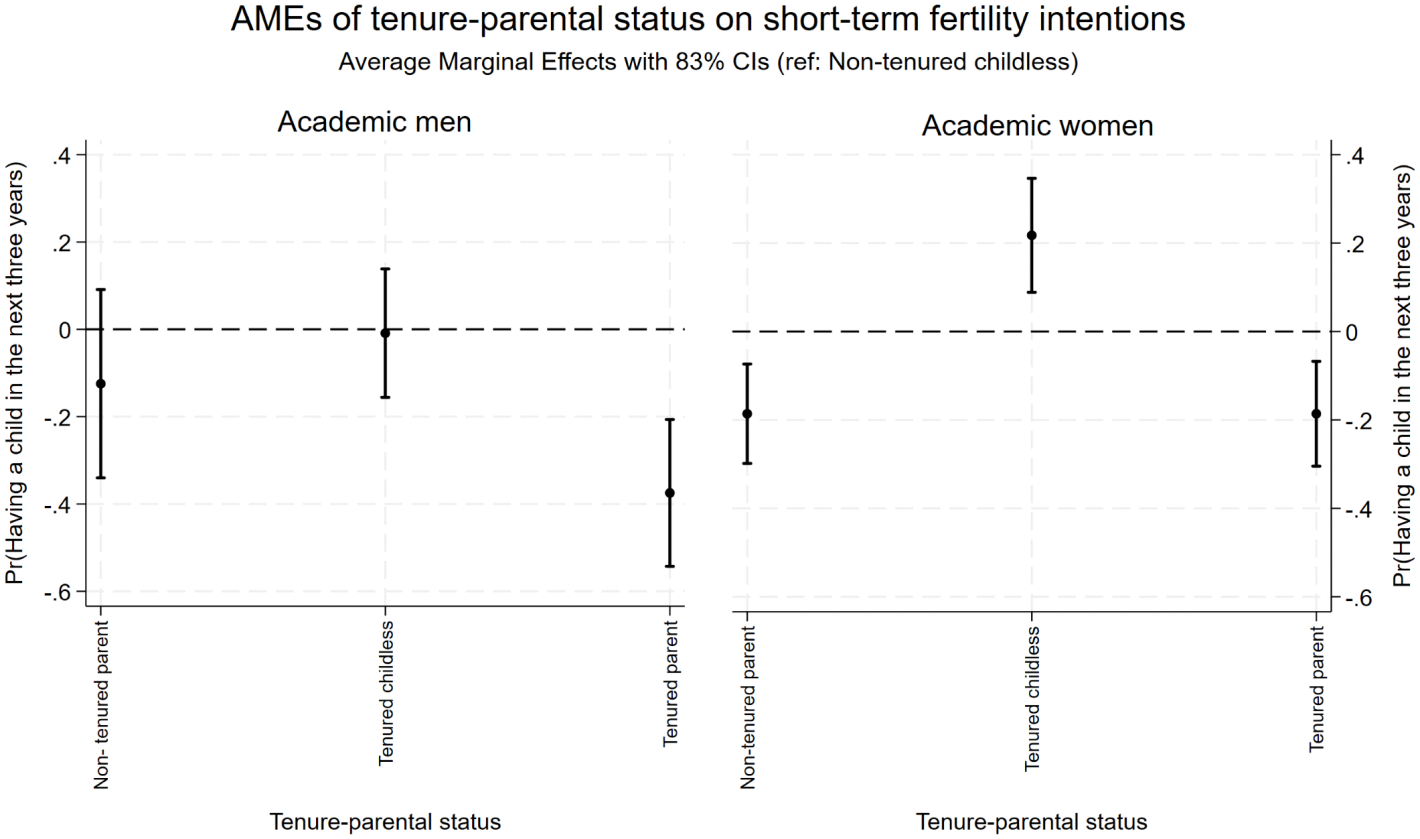
Average marginal effects (AMEs) of tenure-parental status on the short-term fertility intentions among academic men and women: results from Model 5, controlling for partnership, age class, macro region of residence, and field of study.

As reported in Fig 5, attaining a tenured position is positively associated with short-term fertility intentions (Model 5) among childless women (AME = 0.04), while it is independent of tenure among childless men (AME = 0.00). Parents are significantly less likely than childless academics to intend to have another child. As expected, fertility intentions are higher among those who live with a partner and decline with age due to biological constraints to human reproduction. Such a decline, common to both genders, starts earlier for women than for men. Slight variations are observed across geographical areas for women and across scientific disciplines for men (Supporting Information, Table S7 in S1 File).

## Discussion

This paper is the first to jointly examine transitions to first and second childbirth, as well as fertility intentions, among women and men in academia. We find that, as in the general population, academic women initiate both first and second childbirths earlier than academic men. Attaining tenure is an important predictor of the timing of first birth for both men and women. By contrast, tenure does not appear to be related to the transition to a second child. Holding a tenured position also boosts short-term fertility intentions among childless women. No comparable association is observed among men.

Our findings show that men—and, to a lesser extent, women—tend to postpone first-time parenthood until after securing tenure, a milestone that enhances financial stability and access to credit in Italy. In this respect, early-career academics face pressures similar to those experienced by other highly educated professionals navigating prolonged precarity [10]. Not only the year of promotion, but also the following five years are particularly salient for fertility, with a high proportion of academic men and an even higher proportion of academic women transitioning to parenthood during this period. Thus, tenure effectively opens a window of opportunity for fertility among academics, but this window narrows rapidly with age, particularly among women, and eventually closes.

The gender gap in the timing of parenthood found in our sample may reflect anticipatory strategies shaped by asymmetric caregiving burdens. For women, motherhood often entails a sequence of career-disrupting events—pregnancy, childbirth, maternity leave, and early caregiving—that tend to affect research productivity more than fatherhood does for men. Faced with these structural constraints, a higher share of women than men may delay childbirth even after having attained tenure, until their professional identity in the new role is more established, or to mitigate potential motherhood penalties [49,50].

Biological constraints further compound these pressures: as maternal age increases, the probability of conceiving declines, and the time to conception typically lengthens. Together, strategic timing and biological constraints likely contribute to the observed delay in the transition to parenthood and in higher rates of childlessness among women compared to men. Academic men, who generally face fewer direct career costs from parenthood, are less constrained by these dynamics and more likely to time family formation in line with career milestones.

The fact that men’s transition to fatherhood often coincides with achieving tenure may reflect a diverse temporal dynamic. Given the length of academic hiring procedures in Italy, candidates are typically informed of their selection several months before officially taking up the position, which may allow for an anticipatory planning of childbirth. This alignment may also be driven by the increase in income and financial stability that accompany tenure, which can facilitate the transition to parenthood. However, this anticipation effect is not observed among women. It is possible that institutional cultures implicitly perceive impending fatherhood more favorably or normalized than impending motherhood [49], at least upon attaining tenure, subtly shaping career evaluations—though this hypothesis warrants further investigation.

Tenure does not exert a comparable influence on the timing or likelihood of a second birth. This finding suggests that, beyond attaining professional stability, other factors become increasingly salient for expanding one’s family. Childcare availability, the division of household labor, partner support, family orientation, and broader social networks may play a more decisive role in shaping second-birth transitions [51,52]. These factors reflect the intensifying practical and emotional demands of managing work and a larger family.

Holding a tenured appointment strengthens the short-term fertility intentions of childless women, highlighting job security as a key precondition for entering motherhood. The mechanism appears to extend beyond relief from economic uncertainty: achieving tenure also marks an important career milestone, signaling that further advancement is compatible with family formation, so financial stability and professional validation jointly shape women’s family planning. We find no comparable pattern among men, which is consistent with evidence that men’s fertility intentions may be influenced more by their partners’ work–family conflict than by their own [53,54].

According to existing literature, academic women generally exhibit lower fertility levels than their male counterparts: they are more often childless and have fewer children on average. This pattern likely reflects both societal and personal pressures to complete childbearing within a constrained reproductive window, compounded by structural barriers in academia that disproportionately affect women. Given that both biological and institutional timelines for childbearing are more restrictive for women, delays in childbirth may ultimately reduce their completed fertility. These disparities point to broader challenges to fertility within academia [14,55], likely driven by institutional rigidity and a persistent underestimation of biological constraints on fertility. While the trend toward smaller family size mirrors broader societal changes, prior studies show that voluntary childlessness remains rare among academic women, most of whom express a desire for at least two children [56, for Austria].

We acknowledge some limitations. Although post-stratification weights help align our non-probability sample with known population marginal distributions, they cannot guarantee full representativeness, as survey participation may still depend on unobserved factors correlated with our main variables of interest [57,58]. In our case, it is plausible that some individuals decided to participate because they were particularly concerned with the family–career interplay or had salient experiences related to this topic (for instance, having had children after obtaining tenure, or not having children due to job instability). Such respondents might have felt particularly engaged with the survey topic. Conversely, individuals with sufficient time to complete the questionnaire might have been more likely to participate, potentially underrepresenting researchers with children who face greater time constraints due to competing work and family responsibilities. Consequently, it is reasonable to assume that no single, homogeneous group (e.g., academics with children) is overrepresented in our sample. Despite this, our weighting procedure improves correspondence with the reference population, but residual selection biases cannot be completely ruled out [59].

In sum, our study underscores how the early career phase in academia overlaps with women’s prime reproductive years, making the pursuit of tenure and motherhood particularly challenging. Crucially, we also find that career timing matters for men: the likelihood of first childbirth increases once academic men attain tenure, suggesting that men, too, adjust fertility decisions according to their career schedule. Gender gaps appear to stem largely from unequal structural opportunity between women and men rather than from differing preferences. Men continue to reach tenure and other career milestones earlier and more frequently than women [13,60], enabling them to take advantage of the fertility-enhancing effects of career stability. At the same time, men—and often their (younger) partners—remain in their peak reproductive years for longer after attaining tenure than women —and their (older) partners—do. Women, by contrast, typically achieve comparable stability only after a substantial portion of their fertile window has passed, leaving limited time to translate professional stability into realized fertility.

These findings suggest that a reflection on the gendered gap between desired and realized fertility in academia is urgent. Creating accelerated pathways to tenure appears to be a precondition for the transition to parenthood. Other policies, such as extending comprehensive parental support to non-tenured academics, may also help mitigate the intertwined disparities in academic career progression and fertility decisions.

## Supporting information

S1 File Supporting Information for the study. This file contains supporting text (comments on Tables S4 and S6 and Fig S5, questionnaire), Supporting Figures S1–S5, and Supporting Tables S1–S8.
(PDF)
