# Supplementary material for: The best time to become a parent: The relationship between tenure and fertility among academics
Source: PLoS One. 2026 Aug 12;21(8):e0353232. doi: 10.1371/journal.pone.0353232 (PMC13466013; doi:10.1371/journal.pone.0353232)
Supplement: Supplementary file 1 [file pone.0353232.s001.pdf]

Supporting Information
The Best Time to Become a Parent: The Relationship between Tenure and Fertility Among Academics

Supporting Information Text

Comments on Table S4

We include an interaction between tenure and age class in the analysis (Tab. S4). Our findings confirm that, for women, age strongly moderates the tenure effect. Independently of whether they obtained tenure or not, women who are close to the end of their reproductive ages, i.e., past age 45, have a similarly low hazard of becoming mothers and are both significantly less likely to become mothers compared to the reference category, i.e., non-tenured women younger than 35 years old (OR=0.013, $p<0.001$ and OR=0.07, $p<0.05$, respectively).

Also, among women aged 40 to 44, the hazard of becoming mothers is independent of tenure; i.e., it is not statistically different from the hazard for non-tenured women younger than 35 (OR=0.84, $p>0.01$; OR=0.76, $p>0.01$, respectively). However, tenure is significantly and positively associated with transitioning to a first conception among younger women. Being tenured increases the hazard of becoming a first-time mother among women aged less than 35 (OR=1.72, $p<0.01$). Among women aged 35-39, those with a non-tenured academic position are as likely as younger, non-tenured women to become mothers (OR=1.30, $p>0.01$), whereas those with a tenured academic position are significantly more likely than younger, non-tenured women to become mothers (OR=1.60, $p<0.01$; AME=0.039).

Similarly, for men, even if tenured, the hazard of becoming a father past age 45 is lower than it is among non-tenured fathers younger than 35 (OR=0.45, $p<0.01$). There are too few non-tenured men aged 45-49 with children in the sample; hence, the corresponding coefficient cannot be estimated. Tenure increases the hazard of becoming a father among academic men aged 40 to 45 years old compared to non-tenured men aged less than 35 years (OR=1.87, $p<0.01$), whereas the hazard of becoming a father is the same among tenured men aged less than 35 years old and those aged 40-45 (OR=1.09, $p>0.01$). Compared to non-tenured men aged less than 35, the hazard of becoming a father increases among academic men in their late 30s for both those who have a tenured position (OR=2.15, $p<0.001$) and those who do not (OR=1.52, $p<0.01$). Although the OR for those with a tenured position is larger than for those without one, the difference is not statistically significant; hence, the transition to parenthood is independent of tenure for academic men aged 35-39 years. Among academic men aged less than 35 years old, tenure significantly increases the hazard of becoming a first-time father (OR=1.76, $p<0.05$). For all age classes and for both women and men, the hazard of becoming a parent among academics with a tenured position is higher than the hazard among those with a non-tenured position, even if the differences are, as mentioned, not always statistically significant.

Comments on Table S6 and Fig S5

Our findings highlight a significant trend across both genders: the likelihood of having a second child diminishes notably after age 40. Specifically, male academics aged 40-44 exhibit a 65% reduction in the hazard of becoming second-time fathers (OR=0.35, $p<0.001$) compared to those aged 35 or younger. This decline steepens for men aged 45-49 (OR=0.16, $p<0.001$). Similarly, the trend persists among women: female academics aged 40-44 experience a 79% decrease in the hazard of

becoming mothers for the second time (OR=0.21, $p<0.001$) compared to their younger counterparts. For women aged 45-49, the probability of becoming second-time mothers is negligible, underscoring a predictable decline in fertility. We do not find any variation across fields of study or PhD cohorts.

Questionnaire

Dear participant,

The purpose of this survey is to explore the life trajectories of scholars in Italy. By completing the questionnaire, you will help gather data for my PhD project, which aims to explore the relationship between family formation and career progression.

- Depending on your life situation, the survey may take about 10-15 minutes.
- Will be more comfortable answering questions if you keep your CV nearby.
- You can interrupt the survey at any time and resume it later.
- If, for some reason, you do not want to answer a particular question, you can skip it and move on to the next one
- If you have already participated in this survey, please do not do it again.

I really appreciate your contribution!
Thank you!

[Q1] Let's start with general questions. Please indicate the main scientific field in which you are currently working:
Please choose only one of the following:
- Mathematical and computer sciences
- Physical sciences
- Chemical sciences
- Earth sciences
- Biological sciences
- Medical sciences
- Agricultural and Veterinary Sciences
- Civil Engineering and Architecture
- Industrial Engineering and Information Systems
- Sciences of Antiquity, Philology, Literature, and Art History
- Historical, philosophical, pedagogical, and psychological sciences
- Legal sciences
- Economics and Statistics
- Political and social sciences

[Q2] Indicate your gender:
Please choose only one of the following:
- Female
- Male
- Other/Prefer not to say

[Q3] How old are you?
Please choose only one of the following
- 26 or less
- …
- 75 or more

[Q4] Where were you born?
Please choose only one of the following:
- Italy
- Another country (please specify which one)

[Q5] Could you indicate the region of Italy where you are currently working?

Please choose only one of the following:
- North
- Center
- South and Islands

[Q6] Now, let's talk about your job situation. Please indicate your current occupational position in academia:
Please choose only one of the following:
- Full professor
- Associate professor
- Full-time assistant professor
- Tenured assistant professor
- Non-tenured assistant professor
- Post-doctoral research fellowship position
- Other

[Q7] Can you indicate the year you obtained this academic position?
Please choose only one of the following:
- 2021
- …
- 1960 or earlier

[Q8] I would like to reconstruct your academic career so far. Could you indicate the year when you were promoted to a given academic position?
Only answer this question if the following conditions are met: Answer was 'Full professor' at question '6 [Q6]' (Now let's talk about your job situation. Please indicate your current occupational position in academia:) Comment only when you choose an answer. Please choose all that apply and provide a comment:
- Post-doctoral research fellowship position
- Non-tenured assistant professor
- Tenured assistant professor
- Full-time assistant professor
- Associate professor

In case you have skipped some position, leave the field empty

[Q9] I would like to reconstruct your academic career so far. Could you indicate the year when you were promoted to a given academic position:
Only answer this question if the following conditions are met: Answer was 'Associate professor' at question '6 [Q6]' (Now let's talk about your job situation. Please indicate your current occupational position in academia:) Comment only when you choose an answer. Please choose all that apply and provide a comment:
- Post-doctoral research fellowship position
- Non-tenured assistant professor
- Tenured assistant professor
- Full-time assistant professor
- Other:

In case if you have skipped some position, leave the field empty

[Q10] I would like to reconstruct your academic career so far. Could you indicate the year when you were promoted to a given academic position:
Only answer this question if the following conditions are met: Answer was 'Full-time assistant professor' at question '6 [Q6]' (Now let's talk about your job situation. Please indicate your current occupational position in academia:) Comment only when you choose an answer. Please choose all that apply and provide a comment:
- Post-doctoral research fellowship position
- Other:

In case you have skipped some position, leave the field empty

[Q11] I would like to reconstruct your academic career so far. Could you indicate the year when you were promoted to a given academic position:
Only answer this question if the following conditions are met: Answer was 'Tenured assistant professor' at question '6 [Q6]' (Now let's talk about your job situation. Please indicate your current occupational position in academia:) Comment only when you choose an answer. Please choose all that apply and provide a comment:

- Post-doctoral research fellowship position
- Non tenured assistant professor
- Other:

In case you have skipped some position, leave the field empty

[Q12] I would like to reconstruct your academic career so far. Could you indicate the year when you were promoted to a given academic position:
Only answer this question if the following conditions are met: Answer was 'Non tenured assistant professor' at question '6 [Q6]' (Now let's talk about your job situation. Please indicate your current occupational position in academia:) Comment only when you choose an answer. Please choose all that apply and provide a comment:

- Post-doctoral research fellowship position
- Other:

In case you have skipped some position, leave the field empty

[Q13] I would like to reconstruct your academic career so far. Indicate the year when you were promoted to a given academic position:
Only answer this question if the following conditions are met: Answer was 'Other' at question '6 [Q6]' (Now let's talk about your job situation. Please indicate your current occupational position in academia:) Comment only when you choose an answer. Please choose all that apply and provide a comment:

- Post-doctoral research fellowship position
- Non-tenured assistant professor
- Tenured assistant professor
- Full-time assistant professor
- Associate professor
- Full professor
- Other:

In case you have skipped some position, leave the field empty

[Q14] Could you indicate the year when you completed your PhD degree?
Please choose only one of the following:

- I do not have a PhD degree
- 2020
- …
- 1956 or earlier

[Q15] Did you obtain your doctorate in Italy or in another country?
Choose one of the following answers. Please choose only one of the following:

- In Italy
- In another country

[Q16] I would like to ask you about your research activity. Please indicate how many scientific articles you have published in the last three years, possibly indicating how many were published in A-class (Fascia A)* journals (if it applies to your discipline).

	Number of articles	Number of A-class articles
In 2020		
In 2019		
In 2018		

*A-class (Fascia A) scientific journals according to ANVUR (Agenzia Nazionale di Valutazione del Sistema Universitario e della Ricerca)

[Q17] Since the start of your career, how many scientific articles have you published?

Please choose only one of the following:

- 0
- 1 - 4
- 5 - 10
- 11 - 20
- 21 - 30
- 31 - 40
- 41 - 50
- 50 or more

[Q18] Since the start of your career, how many of your scientific articles have been published in the A-class (Fascia A)* scientific journals?

Only answer this question if the following conditions are met: Answer was 'Civil Engineering and Architecture ' or 'Sciences of Antiquity, Philology, Literature and Art History' or 'Historical, philosophical, pedagogical and psychological sciences' or 'Legal sciences' or 'Economics and Statistics ' or 'Political and social sciences' at question '1 [Q1]' (Let's start with general questions. Please indicate the main scientific field in which you are currently working:)

Please choose only one of the following:

- 0
- 1 - 3
- 4 - 6
- 7 - 9
- 10 - 12
- 13 - 14
- 15 - 20
- 21 or more

**A-class (Fascia A) scientific journals according to ANVUR (Agenzia Nazionale di Valutazione del Sistema Universitario e della Ricerca)*

[Q19] For your whole career, how many books have you published?

Only answer this question if the following conditions are met: Answer was 'Civil Engineering and Architecture ' *or* 'Sciences of Antiquity, Philology, Literature and Art History' *or* 'Historical, philosophical, pedagogical and psychological sciences' *or* 'Legal sciences' *or* 'Economics and Statistics ' *or* 'Political and social sciences' at question '1 [Q1]' (Let's start with general questions. Please indicate the main scientific field in which you are currently working:)

Please choose only one of the following:

- 0
- 1
- 2
- 3
- 4
- 5
- 6
- 7
- 8 or more

[Q20] As of today, your H-index is:

Please choose only one of the following:

- 2 or fewer
- 3 - 5
- 6 - 8
- 9 - 11
- 12 - 14

- 15 - 17
- 18 - 20
- 21 or more

[Q21] Have you tried to obtain the national scientific qualification (ASN) in your field?
Only answer this question if the following conditions are met: Answer was greater than or equal to 'Full-time assistant professor' at question '6 [Q6]' (Now let's talk about your job situation. Please indicate your current occupational position in academia:) Please choose only one of the following:

- Yes
- No

[Q22] Did you obtain the national scientific qualification (ASN)?
Only answer this question if the following conditions are met: Answer was 'Yes' at question '21 [Q21]' (Have you tried to obtain the national scientific qualification (ASN) in your field?)
Please choose only one of the following:

- Yes
- No

[Q23] In your own estimation, has your scientific productivity changed during the pandemic (after March 2020)?
Choose what best characterizes you.
Please choose only one of the following:

- I have become more productive in terms of publishing
- Overall my scientific productivity has not changed
- I have become less productive in terms of publishing

[Q24] Please estimate how many hours a week you usually devote to your work. Sometimes, academic working hours vary considerably throughout the year. Give the estimate of your last academic semester.
Please choose only one of the following:

- 50 or more
- …
- 20 or less

"Work" includes all research, teaching, and administrative activities
[Q25] Imagine your usual weekly academic life of the last semester. How many hours do you usually spend on:

Teaching activity (preparing and holding lectures, supervising, etc.)	
Research activity	
Other loads (administrative, technical, etc.)	

[Q26] During your last academic semester, how often did you work on weekends?
Please choose only one of the following:

- Never
- Rarely
- Sometimes
- Often
- Other

[Q27] Have you ever spent more than 6 months working abroad during your academic career?
Only answer this question if the following conditions are met: Answer was 'In Italy' at question '15 [Q15]' (Did you obtain your doctorate in Italy or in another country?)
Please choose only one of the following:

- Yes
- No

[Q28] Have you ever had to move to another country or region to get the academic position you want?
Please choose only one of the following:

- Yes

- No

[Q29] Could you indicate the positions for which you had to move to another country or region of Italy with respect to the previous one?
Only answer this question if the following conditions are met: Answer was 'Yes' at question '28 [Q28]' (Have you ever had to move to another country or region to get the academic position you want?) Check all that apply.
Please choose all that apply:
- Full professor
- Associate professor
- Full-time assistant professor
- Tenured assistant professor
- Non-tenured assistant professor
- Post-doctoral research fellowship position
- Other:

[Q30] Now let's talk about your family situation. What best describes your current marital status?
Please choose only one of the following:
- Married, cohabiting with spouse
- Civilly united (cohabiting with a partner)
- Single
- Married, not cohabiting with spouse (includes de facto separated persons)
- Divorced
- Widowed
- Other

[Q31] How old is your partner?
Only answer this question if the following conditions are met: Answer was 'Married cohabiting with spouse' or 'Civilly united (cohabiting with a partner)' at question '30 [Q30]' (Now let's talk about your family situation. What best describes your current marital status?)
Please choose only one of the following:
- 25 or less
- 26 - 35
- 36 - 45
- 46 or more

[Q32] What year did you start living together with your current partner?
Only answer this question if the following conditions are met: Answer was 'Married cohabiting with spouse' or 'Civilly united (cohabiting with a partner)' at question '30 [Q30]' (Now let's talk about your family situation. What best describes your current marital status?)
Please choose only one of the following:
- Prefer not to answer
- 2021
- …
- 1960 or earlier

[Q33] Which of the following categories best describes your partner's main occupation?
Only answer this question if the following conditions are met: Answer was 'Married cohabiting with spouse' or 'Civilly united (cohabiting with a partner)' at question '30 [Q30]' (Now let's talk about your family situation. What best describes your current marital status?)
Please choose only one of the following:
- Employed in an academic position
- Employed in a research position outside of academia
- Employed in a non-research position
- Self-employed
- Unemployed or not employed
- I do not know
- Other

[Q34] Please indicate your partner’s current position in academia:

Only answer this question if the following conditions are met: Answer was 'Employed in an academic position' at question '33 [Q33]' (Which of the following categories best describes your partner's main occupation?)
Please choose only one of the following:

- Full professor
- Associate professor
- Full-time assistant professor
- Tenured assistant professor
- Non-tenured assistant professor
- Post-doctoral research fellowship position
- I do not know
- Other

[Q35] Is your partner on a full- or part-time contract?
Only answer this question if the following conditions are met:
-------- Scenario 0 --------
Answer was 'Employed in a research position outside of academia' at question '33 [Q33]' (Which of the following categories best describes your partner's main occupation?)
-------- or Scenario 1 --------
Answer was 'Employed in a non-research position' at question '33 [Q33]' (Which of the following categories best describes your partner's main occupation?)
Choose one of the following answers. Please choose only one of the following:

- Full-time contract
- Part-time contract
- I do not know

Full-time employment means a full working day and a working week exceeding 40 hours; part-time is defined as a contract equivalent to 80% or less of a full-time contract.
[Q36] Is your partner on a permanent/open-ended or fixed- term/temporary contract?
Only answer this question if the following conditions are met: Answer was 'Employed in a research position outside of academia' or 'Employed in a non- research position' at question '33 [Q33]' (Which of the following categories best describes your partner's main occupation?)
Choose one of the following answers. Please choose only one of the following:

- Permanent contract
- Fixed-term contract
- I do not know

[Q37] What is the highest qualification level that your partner has obtained?
Only answer this question if the following conditions are met: Answer was 'Married cohabiting with spouse' or 'Civilly united (cohabiting with a partner)' at question '30 [Q30]' (Now let's talk about your family situation. What best describes your current marital status?)

- Please choose only one of the following:
- Primary school or no formal education
- Lower secondary education
- Upper secondary education or vocational education
- Bachelor's degree
- Master's degree or single-cycle
- PhD degree
- I do not know
- Other

[Q38] Do you have children? Please consider also adopted/fostered children.
Please choose only one of the following:

- Yes
- No

[Q39] How many children do you have?
Only answer this question if the following conditions are met: Answer was 'Yes' at question '38 [Q38]' (Do you have children? Please consider also adopted/fostered children.)
Please choose only one of the following:

- 1 child

- 2 children
- 3 children
- 4 children
- 5 children or more

Please consider also adopted/fostered children

[Q40] Does your partner have children (from a previous relationship or adopted children for whom you are not the legal guardian) living in the same house as you?

Only answer this question if the following conditions are met: Answer was 'Married cohabiting with spouse' or 'Civilly united (cohabiting with a partner)' at question '30 [Q30]' (Now let's talk about your family situation. What best describes your current marital status?)

Please choose only one of the following:

- Yes
- No

[Q41] Could you indicate how many of your partner's children (from a previous relationship or adopted children for whom you are not the legal guardian) live in your house?

Only answer this question if the following conditions are met: Answer was 'Yes' at question '40 [Q40]' (Does your partner have children (from a previous relationship or adopted children for whom you are not the legal guardian) living in the same house as you?) Choose one of the following answers

Please choose only one of the following:

- 1 child
- 2 children
- 3 or more children

[Q42] Currently, do you have child(ren) 6 years old or younger living with you?

Only answer this question if the following conditions are met: Answer was 'Yes' at question '38 [Q38]' (Do you have children? Please consider also adopted/fostered children.)

Please choose only one of the following:

- Yes
- No

[Q43] Please indicate the year when your child was born?

Only answer this question if the following conditions are met: Answer was '1 child' at question '39 [Q39]' (How many children do you have?)

[Q44] Please indicate the year when your children were born?

Only answer this question if the following conditions are met: Answer was '2 children' at question '39 [Q39]' (How many children do you have?)

[Q45] Please indicate the year when your children were born.

Only answer this question if the following conditions are met: Answer was '3 children' at question '39 [Q39]' (How many children do you have?)

[Q46] Please indicate the year when your children were born.

Only answer this question if the following conditions are met: Answer was '4 children' at question '39 [Q39]' (How many children do you have?)

[Q47] Please indicate the year when your children were born.

Only answer this question if the following conditions are met: Answer was '5 children or more' at question '39 [Q39]' (How many children do you have?)

[Q48] Can you remember what type of childcare services you used? If you have more than one child, remember your youngest child.

Only answer this question if the following conditions are met: Answer was 'Yes' at question '38 [Q38]' (Do you have children? Please consider also adopted/fostered children.) and Answer was 'No' at question '42 [Q42]' (Currently do you have child(ren) 6 years old or younger living with you?) Check all that apply

Please choose all that apply:

- I did not use any childcare services
- Nursery or infant school
- A babysitter
- Grandparent's support

- Other

[Q49] What type of childcare services do you currently use?
Only answer this question if the following conditions are met: Answer was 'Yes' at question '42 [Q42]' (Currently do you have child(ren) 6 years old or younger living with you?) Check all that apply
Please choose all that apply:
- I do not use any childcare services
- Nursery or infant school
- A babysitter
- Grandparent's support
- Other:

[Q50] Thinking about the total time spent with the child(ren) during the working week, who takes care of the children (cooking, supervision, etc.)?
Only answer this question if the following conditions are met: Answer was 'Single' at question '30 [Q30]' (Now let's talk about your family situation. What best describes your current marital status?) and Answer was 'Yes' at question '42 [Q42]' (Currently do you have child(ren) 6 years old or younger living with you?)
Please choose only one of the following:
- Only me
- Mostly me
- Mostly others
- Other

[Q51] Thinking about the total time spent with the child(ren) during the working week, who takes care of the children (cooking, supervision, etc.)?
Only answer this question if the following conditions are met: Answer was less than or equal to 'Civilly united (cohabiting with a partner)' at question '30 [Q30]' (Now let's talk about your family situation. What best describes your current marital status?) and Answer was 'Yes' at question '42 [Q42]' (Currently do you have child(ren) 6 years old or younger living with you?)
Please choose only one of the following:
- Only me
- Mostly me
- Both my partner and I, equally
- Mostly partner or someone else
- Only partner or someone else
- Other

[Q52] Going back in time to when your children were young and thinking about the total time spent with the child(ren) during the working week, who took care of the children (cooking, supervision, etc.)?
Only answer this question if the following conditions are met: Answer was 'Single' at question '30 [Q30]' (Now let's talk about your family situation. What best describes your current marital status?) and Answer was NOT 'Yes' at question '42 [Q42]' (Currently do you have child(ren) 6 years old or younger living with you?)
Please choose only one of the following:
- Only me
- Mostly me
- Mostly others
- Other

[Q53] Going back in time to when your children were young and thinking about the total time spent with the child(ren) during the working week, who took care of the children (cooking, supervision, etc.)?
Only answer this question if the following conditions are met: Answer was less than or equal to 'Civilly united (cohabiting with a partner)' at question '30 [Q30]' (Now let's talk about your family situation. What best describes your current marital status?) and Answer was NOT 'Yes' at question '42 [Q42]' (Currently do you have child(ren) 6 years old or younger living with you?)
Please choose only one of the following:
- Only me

- Mostly me
- Both my partner and I, equally
- Mostly partner or someone else
- Only partner or someone else
- Other

[Q55] Do you think that your career so far has been affected by the fact of having had children?
Only answer this question if the following conditions are met: Answer was 'Yes' at question '38 [Q38]' (Do you have children? Please consider also adopted/fostered children.)
Please choose only one of the following:

- Yes, mostly positively
- Yes, more positively than negatively
- No, neither positively nor negatively
- Yes, more negatively than positively
- Yes, mostly negatively

[Q56] In assessing the time of the pandemic (after March 2020), has your work-family balance changed from previous years?
Only answer this question if the following conditions are met: Answer was 'Yes' at question '38 [Q38]' (Do you have children? Please consider also adopted/fostered children.)
Please choose only one of the following:

- My work-family balance has not changed
- Since the beginning of the pandemic, I have spent more time on family duties
- Since the beginning of the pandemic, I have spent more time working

[Q57] Would you like to have a (another) child within the next 3 years?
Only answer this question if the following conditions are met: Answer was 'Female' at question '2 [Q2]' (Indicate your gender:) and Answer was less than or equal to '45' at question '3 [Q3]' (How old are you?)
Please choose only one of the following:

- Yes
- No

[Q58] Would you like to have a (another) child within the next 3 years?
Only answer this question if the following conditions are met: Answer was 'Male' at question '2 [Q2]' (Indicate your gender:) and Answer was less than '46 or more' at question '31 [Q31]' (How old is your partner?)
Please choose only one of the following:

- Yes
- No

[Q59] Ideally, how many children in total would you like to have?
Only answer this question if the following conditions are met: Answer was 'Female' at question '2 [Q2]' (Indicate your gender:) and Answer was less than or equal to '45' at question '3 [Q3]' (How old are you?)
Choose one of the following answers. Please choose only one of the following:

- I do not want to have children
- 1 child
- 2 children
- 3 children
- 4 children
- 5 children or more
- Other

Including the number of children you already have (if you have any)

[Q60] Ideally, how many children in total would you like to have?
Only answer this question if the following conditions are met: Answer was 'Male' at question '2 [Q2]' (Indicate your gender:) and Answer was less than '46 or more' at question '31 [Q31]' (How old is your partner?) Choose one of the following answers
Please choose only one of the following:

- I do not want to have children

- 1 child
- 2 children
- 3 children
- 4 children
- 5 children or more
- Other

Including the number of children you already have (if you have any)

[Q61] Could you leave your professional email address or ORCID below?

By providing your email address or your ORCID, you give your consent to access your publication history and link it to your answers to this survey. In so doing, you will allow me to properly investigate the role of family characteristics on the productivity and careers of academics in Italy.

Please write your answer here:

[Q62] Thank you for your participation! If you have any comments or suggestions, you can write them below:

Thank you for your participation!

Supporting Information Figures

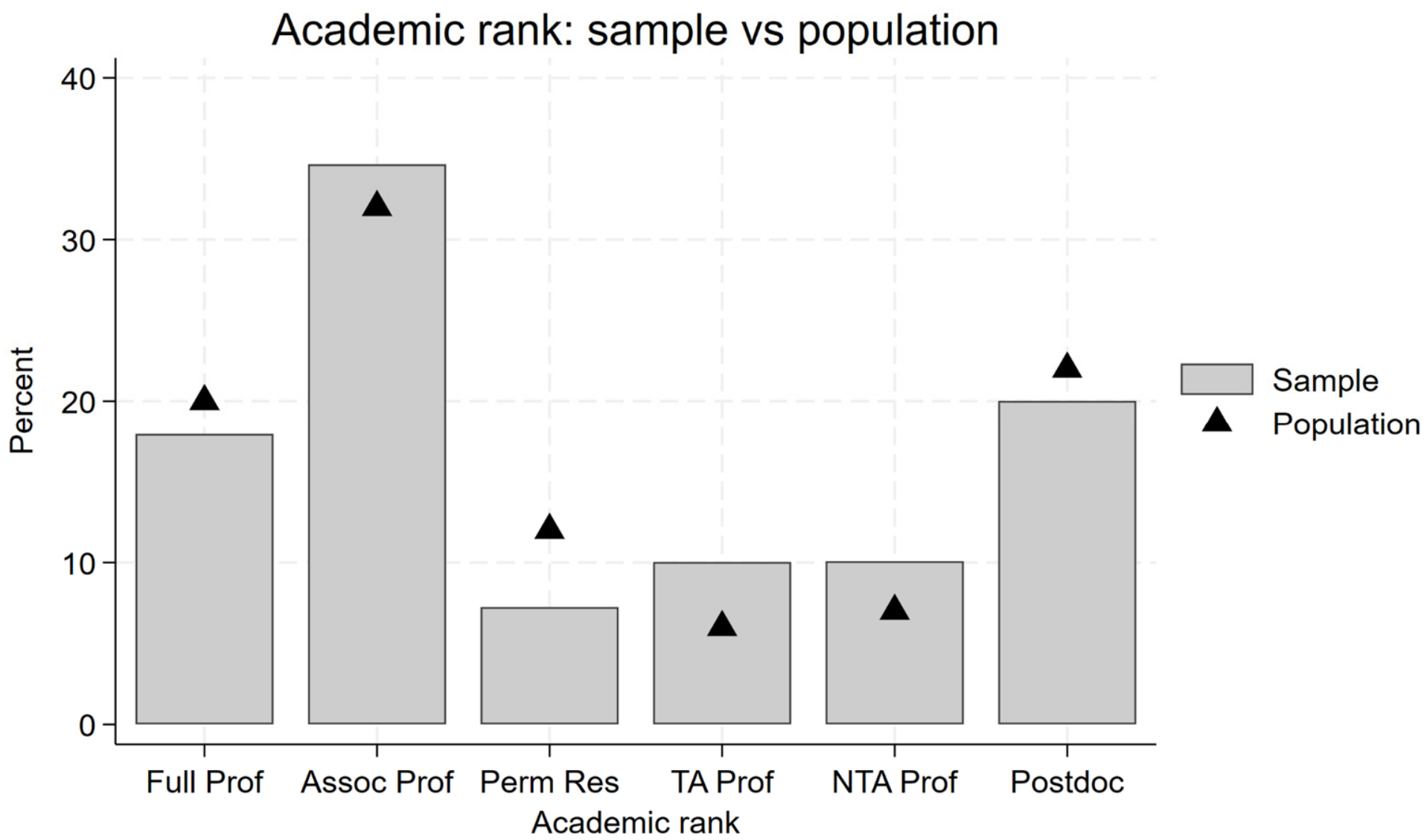

Fig S1. Academic Rank Distribution: Sample (N = 2,262) vs General Population (N = 71,462). *Note: Academic ranks are abbreviated in the x-axis as follows: Full Prof = Full Professor; Assoc Prof = Associate Professor; Perm Res = Permanent Researcher; TA Prof = Tenured Assistant Professor; NTA Prof = Non-Tenured Assistant Professor; Postdoc = Postdoctoral Researcher.*

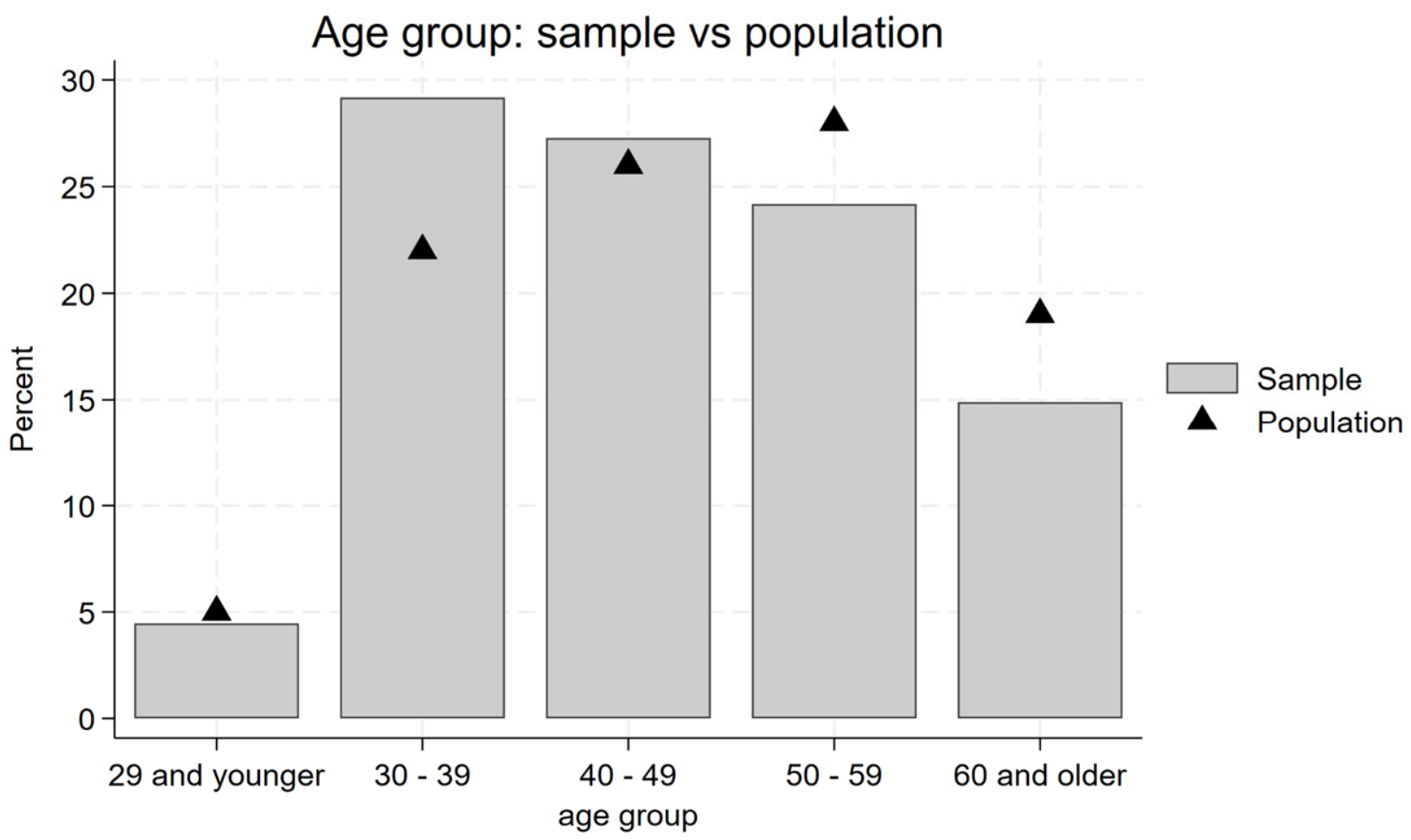

Fig S2. Age Group Distribution: Sample (N = 2,262) vs General Population (N = 71,462)

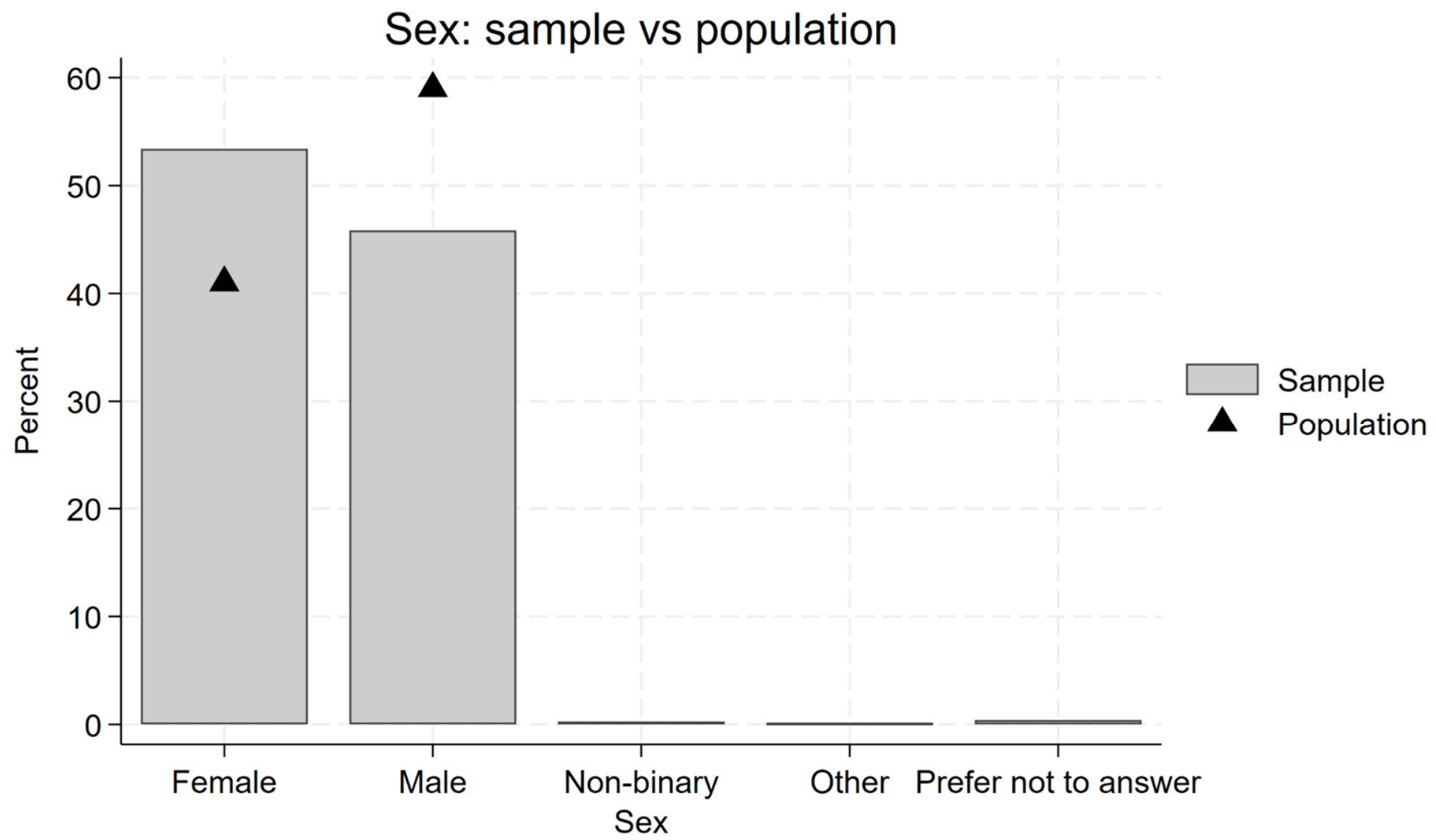

Fig S3. Sex Distribution: Sample (N = 2,262) vs General Population (N = 71,462). *Note: Non-binary, "Other", and "Prefer not to answer" categories are excluded from the analysis because of the very small number of respondents in these groups.*

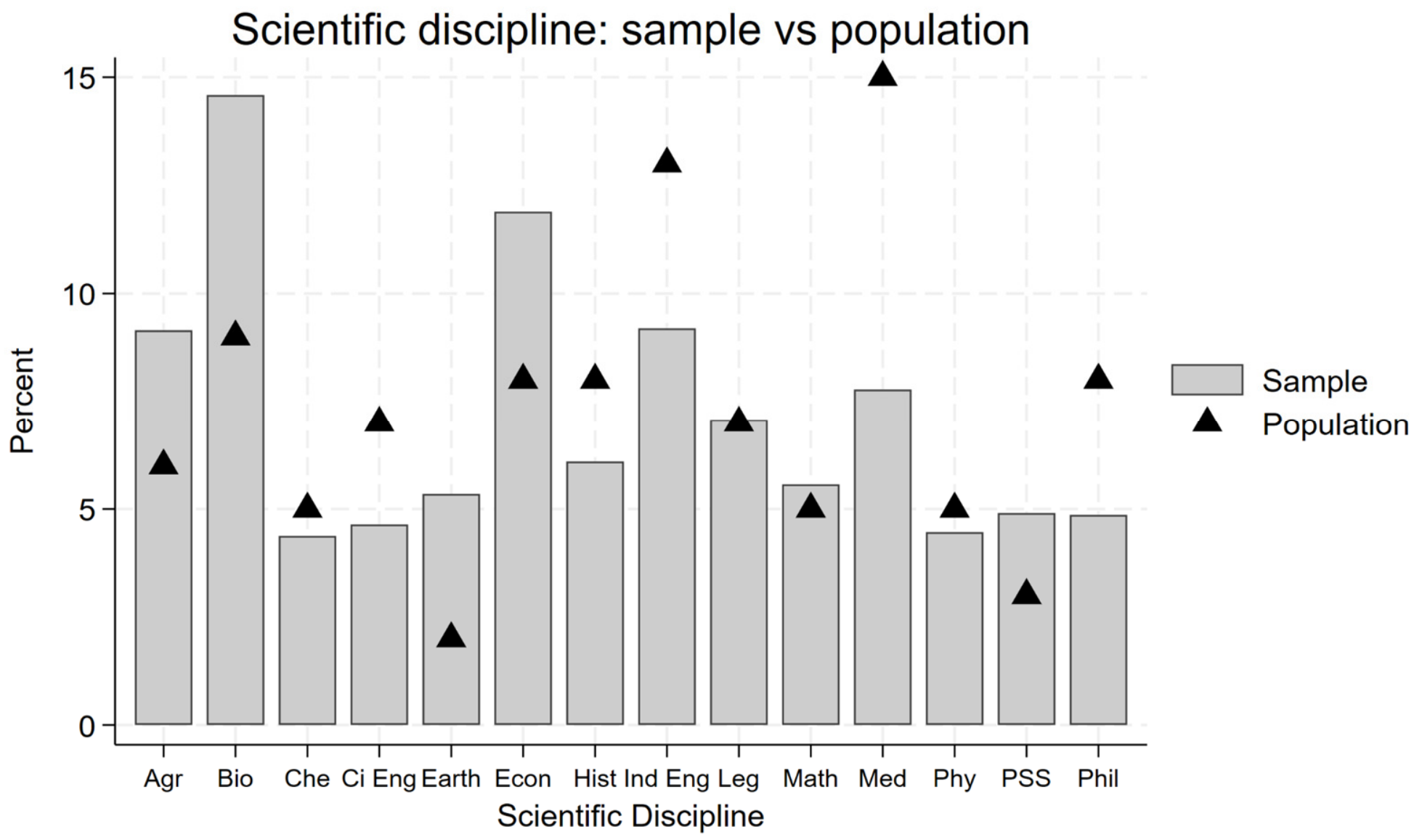

Fig S4. Scientific Discipline Distribution: Sample (N = 2,262) vs General Population (N = 71,462). *Note: Abbreviations on the x-axis denote scientific disciplines as follows: Agr = Agricultural and Veterinary Sciences; Bio = Biological Sciences; Che = Chemical Sciences; Civ Eng = Civil Engineering and Architecture; Earth = Earth Sciences; Econ = Economics and Statistics; Hist = Historical, Philosophical, Pedagogical and Psychological Sciences; Ind Eng = Industrial Engineering and Information Systems; Leg = Legal Sciences; Math = Mathematical and Computer Sciences; Med = Medical Sciences; Phy = Physical Sciences; PSS = Political and Social Sciences; Phil = Sciences of Antiquity, Philology, Literature and Art History.*

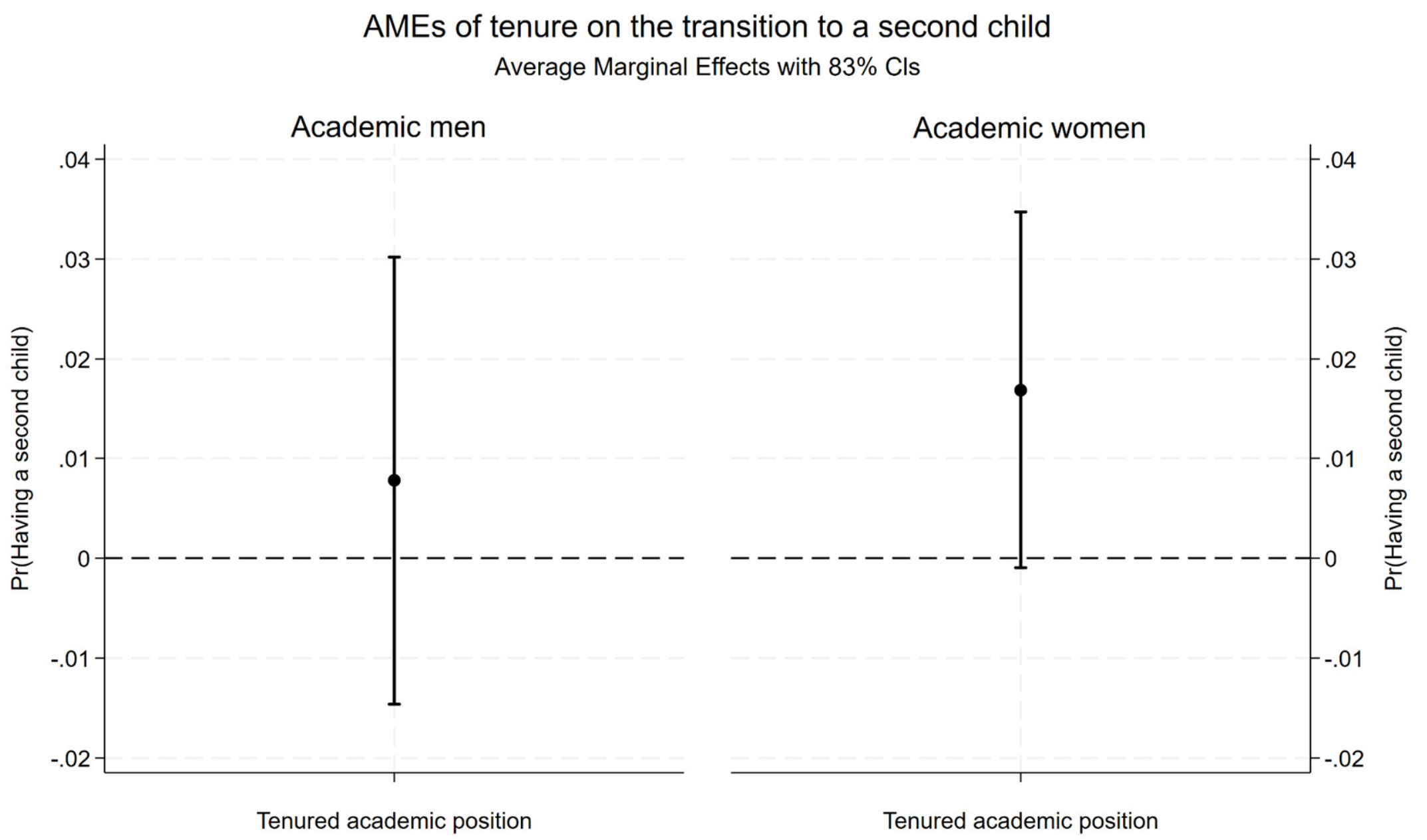

Fig S5. Average marginal effects (AMEs) of tenure versus non-tenure on the probability of having a second child among academic men and women: results from Model 4 controlling for age class, field of study, and PhD cohort.

Supporting Information Tables

Table S1. Description of the samples (time-constant variables only) used for studying the transition to first (Models 1-3) and second (Model 4) child by gender

	Academic Men	Academic Women
**Transitions to 1st child**		
N. persons-years	5,241	5,995
N. obs at risk, among which:	727	876
PhD cohort:		
2009 or earlier	437	516
2010 and more recent	290	360
Field of study		
Natural sciences	321	249
Engineering and Technology	91	131
Health sciences	52	37
Agricultural and Veterinary Sciences	70	75
Social sciences	216	175
Humanities	126	60
**Transitions to 2nd child**		
N. persons-years	2,372	3,105
N. obs at risk, among which:	463	589
PhD cohort:		
2009 or earlier	379	449
2010 and more recent	84	140
Field of study		
Natural sciences	216	157
Engineering and Technology	60	88
Health sciences	37	28
Agricultural and Veterinary Sciences	42	48
Social sciences	160	105
Humanities	74	37

Table S2. Description of the samples used for studying short-term fertility intentions for Model 5, by gender

	Academic Men	Academic Women
Plan to have a (another) child in the next three years	31.16	34.22
Tenure-parenthood status		
Non-tenured childless	19.55	19.25
Non-tenured parents	10.2	21.39
Tenured childless	17.85	16.31
Tenured parents	52.41	43.05
Partnership		
Single/Not living together	29.75	18.18
Living with a partner	70.25	81.82
Age class		
up to 36	15.86	10.16
36 - 40	28.05	44.12
41 and older	56.09	45.72
Macro region of residence in Italy		
Nord	49.01	54.28
Center	30.88	26.2
South and Islands	20.11	18.72
Field of study		
Natural sciences	30.31	34.49
Engineering and Technology	21.53	9.36
Health sciences	3.4	5.35
Agricultural and Veterinary Sciences	8.22	9.89
Social sciences	27.48	27.01
Humanities	9.07	13.9
N.	353	374

Table S3. Age at first and second childbearing, by gender

	Mean	Std. err.	[95% conf.	interval]	N
1st child					
Men	36.90	0.21	36.48148	37.31627	440
Women	35.71	0.18	35.35709	36.05504	502
2nd child					
Men	38.77	0.27	38.23493	39.31303	259
Women	37.99	0.23	37.53325	38.44501	277

Note: Only individuals who had their first and/or second child after completing a PhD are included in the sample.

Table S4. Discrete-time event history regression model on the transition to first child by gender. Results from Model 2.

	Academic Men		Academic Women	
	OR	S.E.	OR	S.E.
Age class## tenured (ref. Younger than 35, Non tenured):				
Younger than 35, Tenured	1.76**	(0.329)	1.72**	(0.296)
35 - 39 Non-tenured	1.52*	(0.286)	1.30	(0.208)
35 - 39 Tenured	2.15***	(0.357)	1.60**	(0.269)
40 - 44 Non-tenured	1.09	(0.434)	0.76	(0.276)
40 - 44 Tenured	1.87*	(0.461)	0.84	(0.175)
45 - 49 Non-tenured	-	-	0.07**	(0.068)
45 - 49 Tenured	0.45*	(0.171)	0.13***	(0.073)
Field of study (ref. Natural sciences):				
Engineering and Technology	1.05	(0.173)	1.27	(0.232)
Health sciences	1.32	(0.277)	1.02	(0.191)
Agricultural and Veterinary Sciences	0.94	(0.188)	0.87	(0.191)
Social sciences	1.10	(0.196)	1.17	(0.159)
Humanities	1.08	(0.223)	0.64*	(0.117)
PhD cohort (ref. 2009 and earlier):				
After 2010	0.99	(0.146)	0.97	(0.135)
Person-years	5,241		5,995	

Note: * p<0.05, ** p<0.01, *** p<0.001.
There are no observations in the male group “45 - 49 Non tenured”.

Table S5. Discrete-time event history regression model on the transition to first-child by gender. Results from Model 3.

	Academic Men		Academic Women	
	OR	S.E.	OR	S.E.
Academic position (ref. Early Career):				
Non-tenured Assistant Professors	2.14**	(0.549)	1.57	(0.393)
Tenured Assistant Professors	1.58**	(0.224)	1.55***	(0.202)
Associate and Full Professors	2.82***	(0.659)	1.10	(0.277)
Age class (ref. Younger than 35):				
35 - 39	1.25	(0.184)	1.12	(0.151)
40 - 44	0.94	(0.230)	0.60**	(0.113)
45 - 49	0.19***	(0.071)	0.10***	(0.051)
Field of study (ref. Natural sciences):				
Engineering and Technology	1.04	(0.173)	1.26	(0.230)
Health sciences	1.36	(0.282)	1.01	(0.188)
Agricultural and Veterinary Sciences	0.93	(0.185)	0.88	(0.192)
Social sciences	1.09	(0.190)	1.19	(0.164)
Humanities	1.08	(0.224)	0.65*	(0.121)
PhD cohort (ref. 2009 and earlier):				
After 2010	0.92	(0.138)	0.92	(0.130)
Person-years	5,241		5,995	

Note: * $p<0.05$, ** $p<0.01$, *** $p<0.001$

Table S6. Discrete-time event history regression model on the transition to a second child by gender. Results from Model 4.

	Academic Men		Academic Women	
	OR	S.E.	OR	S.E.
Tenure status (ref. Non-tenure):				
Tenured	1.08	(0.179)	1.21	(0.176)
Age class (ref. Younger than 35):				
35 - 39	0.74	(0.120)	0.90	(0.124)
40 - 44	0.35***	(0.080)	0.21***	(0.046)
45 - 49	0.16***	(0.050)	0.04***	(0.021)
Field of study (ref. Natural sciences):				
Engineering and Technology	1.49	(0.312)	0.96	(0.199)
Health sciences	1.04	(0.374)	1.11	(0.338)
Agricultural and Veterinary Sciences	1.16	(0.299)	0.65	(0.201)
Social sciences	1.22	(0.254)	0.85	(0.139)
Humanities	1.06	(0.299)	0.87	(0.191)
PhD cohort (ref. 2009 and earlier):				
After 2010	1.40	(0.273)	0.86	(0.170)
Person-years	2,372		3,105	

Note: * p<0.05, ** p<0.01, *** p<0.001

Table S7. Logistic regression on the association between tenure (interaction tenure-having children) and short-term fertility intentions by gender. Results from Model 5.

	Academic Men		Academic Women	
	OR	S.E.	OR	S.E.
Tenure-Parenthood status (ref. Non tenured, childless)				
Non-tenured, parents	0.55	(0.429)	0.37*	(0.163)
Tenured, childless	0.96	(0.496)	2.96*	(1.439)
Tenured, parents	0.12**	(0.085)	0.37*	(0.166)
Marital status (ref. Single/Have a partner, but not living together)				
Living with a partner	5.07**	(2.883)	2.78*	(1.212)
Age class (ref. Younger than 36):				
36 - 40	0.72	(0.363)	0.34*	(0.159)
41 and older	0.26*	(0.142)	0.09***	(0.048)
Macro region of residence (ref. North):				
Center	1.22	(0.429)	2.29*	(0.834)
South and Islands	1.03	(0.394)	1.60	(0.628)
Field of study (ref. Natural sciences):				
Engineering and Technology	1.05	(0.462)	0.75	(0.373)
Health sciences	6.56*	(4.895)	1.08	(0.638)
Agricultural and Veterinary Sciences	3.94*	(2.115)	0.53	(0.250)
Social sciences	1.02	(0.387)	0.68	(0.256)
Humanities	1.29	(0.643)	1.03	(0.495)
N	353		371	

Note: * p<0.05, ** p<0.01, *** p<0.001

Table S8. Sample weights

	Full Professors		*Associate Professors*		*Non-tenured Assistant Professors*		*Tenured Assistant Professors*		*Permanent Researchers*		*Postdoc fellows*	
	M	*F*	*M*	*F*	*M*	*F*	*M*	*F*	*M*	*F*	*M*	*F*
1. Mathematics and informatics	1.35	0.77	1.14	0.81	0.83	0.29	0.84	0.43	2.01	1.27	1.36	0.47
2. Physics	1.50	0.64	0.92	0.57	2.75	0.48	0.95	1.32	***5.28***	1.08	1.43	0.70
3. Chemistry	2.51	1.48	1.92	0.65	0.73	0.55	1.20	1.32	4.96	1.13	2.79	1.07
4. Earth sciences	0.37	0.28	0.49	0.21	0.23	***0.11***	0.88	0.17	0.57	0.26	0.70	0.36
5. Biology	0.83	0.54	0.55	0.44	0.38	0.42	0.68	0.37	1.80	0.99	1.01	0.59
6. Medicine	2.15	1.15	2.53	0.89	4.26	1.43	1.99	0.63	***11.27***	3.57	1.54	1.99
7. Agricultural and Veterinary	0.51	0.77	0.65	0.42	0.41	0.26	0.41	0.52	0.74	0.87	1.31	1.34
8. Civil engineering and architecture	2.48	1.25	1.77	0.94	1.06	0.72	1.00	0.54	4.36	1.37	1.56	1.76
9. Industrial and information engineering	1.21	0.41	1.43	0.85	1.70	0.49	0.71	0.65	1.75	1.90	2.75	2.09
10. Antiquities, philology, literary studies, art history	3.15	1.64	2.95	1.38	-	1.13	1.05	0.69	-	***5.95***	1.02	0.80
11. History, philosophy, pedagogy, and psychology	1.43	1.45	2.33	0.89	2.81	0.58	1.40	0.50	-	1.96	1.28	1.21
12. Law	1.19	0.54	1.48	0.65	0.92	0.70	0.83	0.31	***6.91***	1.35	1.26	2.96
13. Economics and statistics	1.11	0.72	1.10	0.50	0.51	0.51	0.55	0.43	1.75	0.77	0.50	0.40
14. Political and social sciences	-	0.58	1.15	0.80	1.27	0.28	0.43	0.27	0.60	0.94	0.46	0.31
