# Supplementary material for: The best time to become a parent: The relationship between tenure and fertility among academics
Source: PLoS One. 2026 Aug 12;21(8):e0353232. doi: 10.1371/journal.pone.0353232 (PMC13466013; doi:10.1371/journal.pone.0353232)
Supplement: Supplementary file 2 [file pone.0353232.s004.pdf]

Dear Editor,

We would like to thank you for your careful reading of our manuscript and for the very pertinent comments. We also appreciate the opportunity to revise and resubmit our work.

We have revised the text and added further descriptive and contextual information in line with the reviewers' comments. Please find detailed responses to all reviewers' points in the accompanying response letter.

Thank you again for dedicating your time to our work.

Best regards,

The author(s)

Responses to reviewers

Dear Reviewers,

We would like to thank you for your positive assessment of our work and for the thorough reading of our manuscript. We found your comments extremely helpful and have addressed all of them in the revised version.

Below, we provide a detailed account of how we have responded to each of your specific comments and requests. We are confident that the paper has improved thanks to your input.

Thank you again for your constructive feedback.
The author(s)

Response to Additional Editor Comments - p. 2
Responses to Reviewer 1 - pp. 3 - 8
Responses to Reviewer 2 - pp. 9 - 13

Additional Editor Comments

COMMENT: Clarify the precise contribution to the literature. Currently, the authors use imprecise wording such as 'largely overlooked.' Does this mean that this is the first study to investigate both men and women or not? If not, what is the precise contribution? Please avoid this kind of hedging when specifying the contribution of your study.

RESPONSE: Thank you for this comment. We have now clarified the contribution more precisely in the revised manuscript. To the best of our knowledge, no previous quantitative study has jointly examined fertility intentions among male and female academics using comparable measures and analytical strategies. Existing work has overwhelmingly focused on women, typically treating men as a residual category or excluding them altogether. For this reason, we now describe our contribution as ***filling a gap in the literature that has overwhelmingly focused on female academics***, which reflects the state of the field more accurately and avoids unintended ambiguity.

RESPONSES TO REVIEWER 1

COMMENT 1: Line 155-156: Please describe in more detail in the text how your sample deviates from the population. E.g., more women, in which academic rank, in which field,…

RESPONSE: We thank the Reviewer for this suggestion. In the revised version of the manuscript, we have incorporated a detailed comparison between the sample and the target population in the Data Collection section (lines 169–176) and reported the results in a graph in the supporting information (Figure S1-S4). Please find below the text version.

A comparison between the target population and the final sample shows that their composition is broadly similar in terms of sex, academic rank, age, and disciplinary area, with only a few deviations exceeding five percentage points. Women are overrepresented in the sample by 13 percentage points (54% vs. 41%), and academics aged 30–39 by 7 percentage points. Permanent researchers are slightly underrepresented by 5 percentage points. In terms of scientific disciplines, there are no significant deviations, except for scholars in biology, who are overrepresented by 6 percentage points, and those in medicine, who are underrepresented by 7 percentage points.

COMMENT 2: Line 157-156: The authors argue that "To correct for differential participation and ensure population representativeness, we applied direct post-stratification weights." However, weighting alone cannot ensure population representativeness, as participation in the survey may still depend on unobserved factors that are correlated with your main measures of interest. For example, academics who are particularly concerned about the family–career interplay or who have salient experiences (such as having had children after tenure or not having children because of unstable positions) may be more likely to respond than those for whom these issues are less central. Such self-selection cannot be corrected through post-stratification, which adjusts only for observable characteristics. The resulting estimates should therefore be interpreted as weighted associations within the respondent sample rather than as population-level effects. The authors should acknowledge this limitation explicitly and discuss how it might influence interpretation—for instance, whether their findings could represent an upper or lower bound of the associations one would expect in the general population.

RESPONSE: We thank the Reviewer for this comment. We agree that self-selection may be a limitation of our study. Indeed, the deviations between the population and the sample described in the previous point raised by the Reviewer support this limitation, to some extent. For this reason, in the Discussion section (lines 428-442), we explicitly addressed the possibility of self-selection in the sample and highlighted how this may represent a limitation for this type of empirical study. Please find below the text version.

We acknowledge some limitations. While we applied post-stratification weights to align our non-probability sample with the known population margins for key sociodemographic variables, thereby reducing observable discrepancies between our respondents and the target population, weighting alone cannot

ensure full population representativeness, since survey participation may still depend on unobserved factors correlated with the main variables of interest [51, 52]

In our case, it is plausible that some respondents decided to participate because they were particularly concerned about the family–career interplay or had salient experiences related to it (for instance, having had children after obtaining tenure, or not having children due to job instability). Such respondents might have felt especially engaged with the topic of the survey. Conversely, those with sufficient time to complete the questionnaire might have been more likely to participate, potentially excluding researchers with children who face stronger time constraints due to competing work and family demands. Consequently, it is reasonable to assume that not only one single, homogeneous group (e.g., academics with children) is overrepresented in our sample. Despite this, whilst our weighting procedure improves the correspondence with the reference population, residual selection biases cannot be entirely ruled out [53].

COMMENT 3: Model 1 but also other models: is 'younger than 35' the most meaningful cutoff? This may already be late in the fertility timeline. Can you show the distribution of age at parenthood among female and male academics in your sample?

RESPONSE: We believe that this cutoff is meaningful for our sample. Since the risk set for the transition to parenthood consists of individuals who are childless at the time of PhD completion, with time at risk beginning in the year they complete their PhD, we exclude from the models all individuals who became parents before or during PhD completion (who, on average, had their first child before age 29, the mean age at PhD completion in our sample). As a result, the median age at first birth in our analytical sample is equal to 37 for men and 35 for women. The Table below reports the distribution and descriptive statistics of the age at first birth for women and men in the analytical sample.

Also, age 35 is the clinical threshold at which a first-time pregnancy is classified as advanced maternal age, with all the additional screenings and guidelines this entails (Schummers et al., 2018). This threshold is also widely used in the demographic literature to define advanced maternal age (Sobotka & Beaujouan, 2018).

Table 1. Descriptive statistics of age at first childbirth among academic mothers and fathers in the analytical sample.

Female academics				
	Percentiles	Smallest		
1%	29	27		
5%	30	28		
10%	31	29	Obs	502
25%	33	29	Sum of wgt.	346.909998

50%	35		Mean	35.70606
		Largest	Std. dev.	3.984204
75%	38	47		
90%	41	48	Variance	15.87388
95%	44	49	Skewness	0.6694033
99%	46	51	Kurtosis	3.321194
Male academics				
	Percentiles	Smallest		
1%	29	27		
5%	31	28		
10%	32	29	Obs	440
25%	34	29	Sum of wgt.	522.330001
50%	37		Mean	36.89888
		Largest	Std. dev.	4.461349
75%	40	52		
90%	43	54	Variance	19.90364
95%	44	57	Skewness	0.7726274
99%	49	59	Kurtosis	4.232883

COMMENT 4: Lines 206–211: How is the attainment of the National Scientific Abilitation (ASN) treated in your analysis? Following the Gelmini Reform, obtaining the ASN has become a formal prerequisite for tenure in Italy and may itself constitute a significant step toward career stability and family formation. Relatedly, it was unclear to me whether the "attainment of a tenured academic position" on line 206 referred to the actual promotion date, the date of eligibility (i.e., when the ASN was obtained), or the start date of a tenure-track position (e.g., the beginning of an RTD-B research contract). It would be interesting to replicate the analyses using ASN attainment as the key explanatory variable, to see whether this also affects fertility transitions. If the data does not allow it, it would be useful to acknowledge it in the discussion.

RESPONSE: We thank the Reviewer for this insightful comment. We did not collect information on the year in which academics obtained their National Scientific Abilitation (ASN). The "attainment of a tenured academic position" refers to the actual year of promotion to a tenured post, not to the ASN eligibility date nor to the start of a tenure-track contract. We agree that using ASN attainment as a key explanatory variable would be interesting, but this would require both having ASN dates and restricting the analysis to younger cohorts affected by the Gelmini Reform. Following the Reviewer's suggestion, we now mention in

the discussion that future research may consider the association between attaining ASN eligibility -beyond actual promotion- and fertility.

COMMENT 5: I think Models 2 and 3 should be shown in the main text, either as Tables or Figures. To save space, Figure 1 could be moved to the Appendix.

RESPONSE: Thank you, we agree. We have included Models 2 and 3 in the main text as figures:

Figure 3. Average marginal effects (AMEs) of age-tenure group on the probability of having a first child among academic men (left) and women (right): results from Model 2 controlling for field of study and PhD cohort. *Note: There are too few non-tenured men aged 45–49 who become parents in the sample; hence, the corresponding coefficient cannot be estimated, and this interaction term is omitted.*

Figure 4. Average marginal effects (AMEs) of academic position on the probability of having a first child among male (left panel) and female (right panel) academics: results from Model 3 controlling for age class, field of study, and PhD cohort. *Note: NTA-Prof = Non-Tenured Assistant Professor; TA-Prof = Tenured Assistant Professor; A/Full-Prof = Associate or Full Professor.*

COMMENT 6: One final general comment that I have is that to compare associations between men and women, the authors should calculate average marginal effects, given that odds ratios that are estimated in different models cannot be directly compared with each other (Mood 2010). So, statements like the one in the abstract (tenure significantly increases the hazard of becoming a parent for both men and, albeit to a lesser extent, for women) or the one in the conclusion (Our findings show that men—and to a lesser extent women—tend to postpone first-time parenthood until after securing tenure), if based on results of Model 1 (and thus comparing OR=1.62 for men and OR=1.44 for women), should be backed by calculations of Average Marginal Effects.

RESPONSE: We thank the Reviewer for this insightful comment. In response, we have calculated Average Marginal Effects (AMEs) to compare the associations between men and women more accurately, as recommended. We have included these results in the main text (Figures 2-5), while maintaining the Odds Ratios (ORs) in the regression tables for consistency with the original model output. Additionally, we have edited the text to explicitly reference the significance of the comparisons based on AMEs. Please, find the description of the first model below:

The results of Model 1 (Tab.1) show that after controlling for age, PhD cohort, and field of study, tenure significantly increases the hazard of becoming a parent. For academic men, the hazard of conceiving a child in the years following promotion increases by 62% compared to non-tenured men (OR = 1.62, p < 0.001), and for academic women, by 44% compared to non-tenured women (OR = 1.44, p < 0.01). However, when comparing the average marginal effects (AMEs) of tenure on the probability of having a

first child, the effect is stronger for men: the AME for academic men is 0.04, compared to 0.03 for academic women (Fig. 2).

Minor comments

- Lines 84-87: improve phrasing, it is unclear which of the themes is underexplored

RESPONSE: Thank you, we have now edited the sentence to improve clarity and, following also the Editor's comments, we added a new sentence clarifying the contribution to previous literature (lines 84-93)

While research has extensively documented the parental penalties faced by academic mothers, the fertility trajectories of academic men, the impact of fatherhood on their academic careers, and the ways in which career advancement influences men's reproductive outcomes remain underexplored. To date, no previous quantitative study has jointly examined and compared fertility intentions among male and female academics. Existing literature has overwhelmingly focused on women, often treating men as a residual category or excluding them altogether. However, emerging cohorts of academic fathers are experiencing increasing parenting demands as social norms shift toward greater paternal involvement in childcare. Despite this, academic men continue to have higher completed fertility rates than their female colleagues, and fatherhood is not typically associated with declines in their scholarly output

- Line 110-111: specify what you mean by faculty position, who is it that you are dropping?

RESPONSE: We have now edited the original sentence to improve clarity (lines 129-133):

The survey explicitly excluded PhD holders who had left academia before 2022. We also excluded individuals employed in non-standard academic positions within the Italian university system, including doctoral candidates, research grant holders, post-graduate fellows, collaborators on coordinated and continuous contracts, and adjunct professors. These roles fall outside the standard faculty career track and were therefore outside the target population of our study.

- Line 127: "identified through systematic keyword searches". What keyword searches?

RESPONSE: Thank you for the comment, with which we agree. For this reason, we added in the main text the following paragraph (lines 140-152):

The search for scientific associations was carried out in Italian using keywords identifying the type of organisation (e.g., "association", "society"), combined with the names of the 14 scientific-disciplinary areas listed in the MUR open data: "Mathematics and Computer Sciences", "Physical Sciences", "Chemical Sciences", "Earth Sciences", "Biological Sciences", "Medical Sciences", "Agricultural and Veterinary Sciences", "Civil Engineering and Architecture", "Industrial and Information Engineering", "Ancient, Philological-Literary and Historical-Artistic Studies", "Historical, Philosophical, Pedagogical and

Psychological Sciences", "Legal Sciences", "Economic and Statistical Sciences", "Political and Social Sciences". Once the official website of a relevant scientific association was identified, we searched for contact details, typically those of the secretary or the president. In total, we identified 218 national scientific academic associations with available contact information, covering all 14 disciplinary areas. An email describing the study and requesting the dissemination of the survey was sent to these contacts. In the absence of a response within one week, a follow-up email was sent after one and two weeks. As a result, 72 scientific associations distributed the survey.

- Line 166: specify where this share is taken from

RESPONSE: We specified the source in the text (lines 181-182):

The population quotas (defined by gender × academic rank × disciplinary affiliation) were calculated using the official microdata on university personnel released by MUR.

The individual-level administrative data from the Italian Ministry of University and Research (MUR), available through the *Portale dei dati dell'istruzione superiore* (USTAT): https://ustat.mur.gov.it/.

Direct link:
https://dati-ustat.mur.gov.it/dataset/serie-storica-sul-personale-universitario/resource/3bdba958-5c6c-4b4e-a830-70e30014ee94

- Line 269: "with no notable differences between women aged below or above 35.": no differences between women aged below 35 and between 35-39 I suppose.

RESPONSE: Yes, we corrected the text accordingly.

- Lines 32, 301, 315, 353: why 'young' childless women? From Table S7 the reference category is 'non-tenured, childless', and the significant coefficient is for 'Tenured, childless', so you do not define any age category.

RESPONSE: The Reviewer is right, thank you for spotting this mistake. We now deleted the word "young.

- I would rather always frame your findings in terms of "associations" rather than "effects" (see, e.g., line 96), given that you do not use causal methods. Talking about the 'effect' of tenure might be misleading.

RESPONSE: Corrected.

RESPONSES TO REVIEWER 2

COMMENT 1: I lack a clear context of the Italian case. Here are some aspects that I would like more info on: * What do parental leave rights look like in Italy? Are they linked to tenure or do everyone have the same rights on the labor market? How might such rights influence the choice of having children?

RESPONSE: Thanks for your comment. We integrated the following description in the introduction (lines 99-111)

Moreover, in Italy, employed mothers are entitled to five months of paid maternity leave, while employed fathers are entitled to ten days of paternity leave [38]. In addition, employed parents can take up to ten months of parental leave per child, extended to eleven months if the father uses at least three months, until the child's twelfth birthday.

By contrast, doctoral candidates are not standard employees and do not have access to parental leave under the same conditions. Instead, they benefit from the automatic suspension and extension of their doctoral enrolment (and, where applicable, their scholarship) during periods of maternity or paternity leave. Postdoctoral researchers and other early-career academics may either be covered by standard parental leave provisions or by similar suspension mechanisms, depending on their contract type. This institutional framework, combined with the disproportionate use of parental leave by women (63% of mothers versus only 8% of fathers across all employment categories [39]), may result in academic mothers spending longer periods away from work compared to fathers, thereby reinforcing cumulative disadvantages in career continuity and research productivity.

COMMENT 2: It would be useful to understand whether it is common for phd students to have children as it is in Nordic countries. Maybe it is not common, but it could be interesting for the reader, even though this group is not analyzed. If not common, is this because phd students are typically younger in Italy?

RESPONSE: We thank the Reviewer for this interesting point. It is relatively uncommon for PhD students to have children in Italy.

To the best of our knowledge, there are no studies in Italy that track parenthood among PhD students. Administrative data are not publicly available. The only official survey on PhD holders (the ISTAT cohort survey, conducted 4–6 years after PhD completion) does not collect information on the year of childbirth, but only on parental status at the time of the interview. In this sense, our survey is, as far as we are aware, the only (unofficial) data source that contains detailed information on the timing of childbirth relative to PhD completion for both men and women, which we believe is a valuable contribution to the literature.

We report below some descriptive statistics regarding the timing of the first childbirth by gender. Table 1 summarizes the timing of the first childbirth in relation to PhD completion among women and men in our sample, using post-stratification weights.

Parenthood during the PhD competition year is relatively uncommon for both genders. Among women, 19.5% had their first child before completing their PhD, 5.9% during the PhD year, and the majority (74.6%) after PhD completion. The patterns are similar for men, although fewer became parents before completing their PhD (11.0%) and slightly more during the PhD completion year (7.7%), with 81.3% becoming fathers after completing their PhD. It is important to note that, in our models, we define career start as PhD completion (since, nowadays, it is a prerequisite for the standard academic career track). Therefore, individuals who became parents before completing their doctoral studies are excluded from the analysis.

The low incidence of childbirth during the PhD in Italy is consistent with structural characteristics of the doctoral training system. Italian PhD scholarships are relatively modest—often below the level of a living wage—and PhD candidates who are not on a scholarship typically have no income support at all. Moreover, until very recently, parental leave provisions for PhD students were limited: maternity leave existed, but no formal parental leave system comparable to those available to employees, and fathers had almost no dedicated rights. In addition, PhD students are legally classified as students rather than employees, and therefore lack access to the broader social protection system (e.g., paid parental leave, childcare support schemes).

The absence of formal employment status, combined with financial insecurity and the perception that parenting may delay academic progress, contributes to discouraging family formation during doctoral studies in Italy. These structural constraints stand in sharp contrast to Nordic countries, where doctoral students benefit from stronger financial support and more comprehensive parental leave policies, making childbirth during the PhD more common.

Table 2. The timing of the first childbirth (before, during, and after PhD completion year) among female and male respondents (weights are applied).

	Women		Men		Total	
	N	%	N	%	N	%
Before PhD completion	116	19.5%	53	11.0%	169	14.6%
At the PhD completion year	38	5.9%	37	7.7%	75	6.9%
After PhD completion	502	74.6%	440	81.3%	942	78.5%
Total	656		530		1,186	

COMMENT 3: I would also see some descriptive material of the data analyzed including age, position and university affiliation and how this relates to the population of academics.

RESPONSE: Following the Reviewer's comment, in the Data Collection section of the revised version of the manuscript, we now report a detailed comparison between our sample and the target population (lines 169–176), also making use of a graphical representation in the supplementary materials (Figure S1-S4). We provide comparisons by gender, age (in classes), academic rank, and scientific discipline, while information on university affiliation was not collected in the questionnaire due to privacy constraints.

A comparison between the target population and the final sample shows that their composition is broadly similar in terms of sex, academic rank, age, and disciplinary area, with only a few deviations exceeding five percentage points. Women are overrepresented in the sample by 13 percentage points (54% vs. 41%), and academics aged 30–39 by 7 percentage points. Permanent researchers are slightly

underrepresented by 5 percentage points. In terms of scientific disciplines, there are no significant deviations, except for scholars in biology, who are overrepresented by 6 percentage points, and those in medicine, who are underrepresented by 7 percentage points.

COMMENT 4: Data sampling/collection:
1) A response rate of only 3.5% suggests serious risks of sample selection bias. This does not only concern selection on observables, which the authors try to address by weighting, there could also be a risk that non-observables bias the results. For instance, only those with sufficient time to answer will do so. Thus, less busy researchers may answer. Also, those particularly engaged in the topic of the survey may choose to respond. The authors do not discuss this at all.

RESPONSE: We thank the reviewer for having raised this point, which mentioned the other reviewer, too. Accordingly, we discussed this issue in the Discussion section (lines 428-442), pointing to the limitation that our sample might have.

We acknowledge some limitations. While we applied post-stratification weights to align our non-probability sample with the known population margins for key sociodemographic variables, thereby reducing observable discrepancies between our respondents and the target population, weighting alone cannot ensure full population representativeness, since survey participation may still depend on unobserved factors correlated with the main variables of interest [51, 52]

In our case, it is plausible that some respondents decided to participate because they were particularly concerned about the family–career interplay or had salient experiences related to it (for instance, having had children after obtaining tenure, or not having children due to job instability). Such respondents might have felt especially engaged with the topic of the survey. Conversely, those with sufficient time to complete the questionnaire might have been more likely to participate, potentially excluding researchers with children who face stronger time constraints due to competing work and family demands. Consequently, it is reasonable to assume that not only one single, homogeneous group (e.g., academics with children) is overrepresented in our sample. Despite this, whilst our weighting procedure improves the correspondence with the reference population, residual selection biases cannot be entirely ruled out [53].

COMMENT 5: 2) This raises issues of why not alternative data collection procedures have not been undertaken, for instance directly approaching researchers with interviews or using administrative data. The authors should discuss these objections.

RESPONSE: We thank the Reviewer for these important points. Administrative data sources in Italy that provide information on fertility for all academics are unavailable. Existing data cover only individuals who have taken maternity, paternity, or parental leave. The only administrative source used in previous work is restricted to female academics who obtained the National Scientific Habilitation (ASN), as in De Paola, M., Nisticò, R., & Scoppa, V. (2022), *Academic careers and fertility decisions*, in *Teaching, Research and Academic Careers: An Analysis of the Interrelations and Impacts* (Springer). This dataset does not include male academics nor allow us to reconstruct the timing of childbirth in relation to PhD completion and career trajectories for the entire academic population.

Concerning alternative data collection strategies (e.g., directly approaching researchers or conducting interviews), our options were constrained by Italian data protection regulations and the EU General Data Protection Regulation (GDPR; Regulation (EU) 2016/679), which governs the processing of personal data and imposes strict limits on the collection and use of identifiable information for research purposes. In particular, we were not allowed to obtain or use personal contact information (such as institutional e-mail addresses) from administrative registers when there is a risk that individuals could be identified, especially in connection with sensitive information such as fertility and family formation. This prevented us from implementing targeted recruitment strategies based on administrative lists of academics. For the same reasons, conducting large-scale interviews based on identifiable sampling frames was not feasible.

Within these legal and practical constraints, a web-based survey disseminated through professional and academic channels represented the only realistic way to collect detailed, individual-level information on the timing of childbirth for both men and women across academic ranks at the national level.

COMMENT 5: Weighting:
If I understand correctly there were 84 strata by gender, but as written now there are 2 x 6 x 14 = 168 strata. Clarify this point.

RESPONSE: Thank you for noticing this inconsistency; it was our calculation error. There are 168 strata in total (2 genders, 6 academic ranks, and 14 scientific disciplines).

COMMENT 6: Wordings:
126: "two-pronged" rather than "two-prolonged"

RESPONSE: corrected, thank you.

COMMENT 7: 126-129: the distribution of the survey is not clearly described. Were 218 associations contacted and 72 distributed it? Or did you chose them? Similarly, were universities chosen by the authors for distribution, or how was this done?

RESPONSE: We thank the Reviewer for this question. We included more detailed information regarding the Survey distribution. Please find it below and in the corresponding section (lines 139 - 160):

The survey was disseminated through national academic associations and Italian universities.

The search for scientific associations was carried out in Italian using keywords identifying the type of organisation (e.g., "association", "society"), combined with the names of the 14 scientific-disciplinary areas listed in the MUR open data: "Mathematics and Computer Sciences", "Physical Sciences", "Chemical Sciences", "Earth Sciences", "Biological Sciences", "Medical Sciences", "Agricultural and Veterinary Sciences", "Civil Engineering and Architecture", "Industrial and Information Engineering", "Ancient, Philological-Literary and Historical-Artistic Studies", "Historical, Philosophical, Pedagogical and Psychological Sciences", "Legal Sciences", "Economic and Statistical Sciences", "Political and Social Sciences". Once the official website of a relevant scientific association was identified, we searched for contact details, typically those of the secretary or the president. In total, we identified 218 national scientific academic associations with available contact information, covering all 14 disciplinary areas. An email describing the study and requesting the dissemination of the survey was sent to these contacts. In

the absence of a response within one week, a follow-up email was sent after one and two weeks. As a result, 72 scientific associations distributed the survey.

We then relied on the complete list of Italian universities available on the official website of the Ministry of University and Research, which includes 61 public and 20 private institutions. For each university, we consulted the official website to identify relevant contact persons, usually the secretary, the Chair of the Single Guarantee Committee (CUG), or the vice-rector for research. Through this procedure, we identified 74 universities with suitable contact information, which were contacted via e-mail and invited to disseminate the survey among faculty members. As with the scientific associations, non-responses were followed up with two reminder e-mails. In total, 37 universities agreed to distribute the survey among their academic staff.

COMMENT 8: 215: Explain the "Gelmini reform" and how it impacted on academic career trajectories.

RESPONSE: Thank you for pointing this out. We proceeded accordingly, explicating what the Gelmini reform is (lines 255-260 in the Manuscript).

… the Gelmini reform in Italy (Law 240/2010), a law that significantly affected academic career trajectories. This university reform introduced the National Scientific Qualification by disciplinary field, based on standardized criteria set by the MUR, making it a prerequisite for appointment as Associate or Full Professor. The reform also created one junior assistant professorship position, which is non-tenured, and a senior assistant professorship (tenure-track) position.

COMMENT 9: 224: Change "those 52%" to "of which 52%"

RESPONSE: corrected in all cases.

COMMENT 10: 256: "approach" is not the right word here.

RESPONSE: corrected for “birth spacing patterns” instead of approach.
