# Supplementary material for: The best time to become a parent: The relationship between tenure and fertility among academics
Source: PLoS One. 2026 Aug 12;21(8):e0353232. doi: 10.1371/journal.pone.0353232 (PMC13466013; doi:10.1371/journal.pone.0353232)
Supplement: Supplementary file 3 [file pone.0353232.s002.docx]

***“The Best Time to Become a Parent: The Effect of Tenure on Fertility Among Academics”***

**Referee report**

This study examines how tenure attainment is associated with transitions into first and second childbirth, as well as fertility intentions, among Italian male and female academics. The paper shows that the hazard of becoming a parent increases when both male and female academics obtain a tenured position, relative to untenured male and female academics. No significant associations are found between tenure attainment and second childbirth. Finally, among childless female academics, tenure attainment is associated with positive fertility intentions. Overall, I find the topic and paper interesting and relevant, but there are some points I would like the authors to address, as I outline below.

Main comments

- Line 155-156: Please describe in more detail in the text how your sample deviates from the population. E.g., more women, in which academic rank, in which field,…
- Line 157-156: the authors argue that “To correct for differential participation and ensure population representativeness, we applied direct post-stratification weights.” However, weighting alone cannot ensure population representativeness, as participation in the survey may still depend on unobserved factors that are correlated with your main measures of interest. For example, academics who are particularly concerned about the family–career interplay or who have salient experiences (such as having had children after tenure or not having children because of unstable positions) may be more likely to respond than those for whom these issues are less central. Such self-selection cannot be corrected through post-stratification, which adjusts only for observable characteristics. The resulting estimates should therefore be interpreted as weighted associations within the respondent sample rather than as population-level effects. The authors should acknowledge this limitation explicitly and discuss how it might influence interpretation—for instance, whether their findings could represent an upper or lower bound of the associations one would expect in the general population.
- Model 1 but also other models: is ‘younger than 35’ the most meaningful cutoff? This may already be late in the fertility timeline. Can you show the distribution of age at parenthood among female and male academics in your sample?
- **Lines 206–211:** How is the attainment of the National Scientific Abilitation (ASN) treated in your analysis? Following the Gelmini Reform, obtaining the ASN has become a formal prerequisite for tenure in Italy and may itself constitute a significant step toward career stability and family formation. **Relatedly**, it was unclear to me whether the “attainment of a tenured academic position” on line 206 referred to the **actual promotion date**, the **date of eligibility** (i.e., when the ASN was obtained), or the **start date of a tenure-track position** (e.g., the beginning of an RTD-B research contract). It would be interesting to replicate the analyses using ASN attainment as the key explanatory variable, to see whether this also affects fertility transitions. If the data does not allow it, it would be useful to acknowledge it in the discussion.
- I think Models 2 and 3 should be shown in the main text, either as Tables or Figures. To save space, Figure 1 could be moved to the Appendix.
- One final general comment that I have is that to compare associations between men and women, the authors should calculate average marginal effects, given that odds ratios that are estimated in different models cannot be directly compared with each other (Mood 2010). So, statements like the one in the abstract (tenure significantly increases the hazard of becoming a parent for both men and, albeit to a lesser extent, for women) or the one in the conclusion (Our findings show that men—and to a lesser extent women—tend to postpone first-time parenthood until after securing tenure), if based on results of Model 1 (and thus comparing OR=1.62 for men and OR=1.44 for women), should be backed by calculations of Average Marginal Effects.

Minor comments

- Lines 84-87: improve phrasing, it is unclear which of the themes is underexplored
- Line 110-111: specify what you mean by faculty position, who is it that you are dropping?
- Line 127: “identified through systematic keyword searches”. What keyword searches?
- Line 166: specify where this share is taken from
- Line 269: “with no notable differences between women aged below or above 35.”: no differences between women aged below 35 and between 35-39 I suppose.
- Lines 32, 301, 315, 353: why ‘young’ childless women? From Table S7 the reference category is ‘non-tenured, childless’, and the significant coefficient is for ‘Tenured, childless’, so you do not define any age category.
- I would rather always frame your findings in terms of "associations" rather than "effects" (see, e.g., line 96) given that you do not use causal methods. Talking about the ‘effect’ of tenure might be misleading.

References

Mood, C. (2010). Logistic regression: Why we cannot do what we think we can do, and what we can do about it. *European sociological review*, *26*(1), 67-82.
