# Supplementary material for: The best time to become a parent: The relationship between tenure and fertility among academics
Source: PLoS One. 2026 Aug 12;21(8):e0353232. doi: 10.1371/journal.pone.0353232 (PMC13466013; doi:10.1371/journal.pone.0353232)
Supplement: Supplementary file 4 [file pone.0353232.s005.docx]

*Dear Editor, dear Reviewers,*

*We thank you for the careful and constructive evaluation of the revised manuscript.*

*We are grateful for the positive assessment and for your thoughtful comments and suggestions, which have helped us to refine the paper's focus further and improve its overall quality.*

*In addition to the revisions detailed in this response, the manuscript has been extensively revised, as suggested, to improve overall writing quality, including clarity, grammar, and flow. Please find our point-to-point responses below.*

*Thank you again for dedicating your time to our work.*

*Best regards,*

*The authors*

**RESPONSES TO REVIEWER 1**

**Reviewer #1:**

*I am generally satisfied with revisions made by authors to the paper, I only have these remaining comments:*

*• “Existing literature has overwhelmingly focused on women, often treating men as a residual category or excluding them altogether. However, emerging cohorts of academic fathers are experiencing increasing parenting demands as social norms shift toward greater paternal involvement in childcare.”: Can you add references to these sentences/statements?*

**RESPONSE:** Thank you for your suggestion. We have included the following references on academic fathers in the text:

[36] - Cannito, M., & Santero, A. (2024). Balancing work, life, and care responsibilities in academia: the male perspective. *Sociologia del lavoro*, (2024/168). <https://doi.org/10.3280/SL2024-168005>

[37] - Damaske, S., Ecklund, E., Lincoln, A., & White, V. (2014). Male Scientists’ Competing Devotions to Work and Family: Changing Norms in a Male-Dominated Profession. Work and Occupations, 41, 477 - 507. <https://doi.org/10.1177/0730888414539171>.

The edited version of the text now reads as follows (lines 91-94):

“The existing literature has overwhelmingly focused on women, often treating men as a residual category or excluding them altogether, with only sparse, dated exceptions [8]. However, emerging cohorts of academic fathers are experiencing increasing parenting demands as social norms shift toward greater paternal involvement in childcare [36, 37, 6].

*• Lines 309-311: is this difference between men and women statistically significant? Can you comment more on how large this difference is (also in the discussion)?*

**RESPONSE:** The difference between men and women is not statistically significant, as shown by the overlapping confidence intervals of the AMEs in Figure 2. We have added a comment on this point in the Results section at lines 317-319:

“The average marginal effects (AMEs) of tenure on the probability of having a first child are slightly higher for academic men than women (but the difference is not statistically significant because of overlapping CIs; see Fig. 2).”

*• While going over the paper again, I noticed you always have 83% confidence intervals in the figures. Why this choice? I would suggest using standard 95% confidence intervals.*

**AUTHORS' RESPONSE:** We use 83% rather than 95% confidence intervals in the figures to facilitate the visual comparison of marginal effects. Previous studies (e.g., Bolsen & Thornton, 2014; Goldstein & Healy, 1995; Knol et al., 2011) demonstrated that, when comparing two estimates, nonoverlapping 83% CIs correspond approximately to a statistically significant difference at the 5% level. Using 83% intervals, therefore, allows readers to assess whether differences in predicted probabilities are statistically meaningful visually. For clarity, we have now added the following sentence in the paper (lines 293-296):

“All figures reporting average marginal effects (Figs 2-5) use 83% confidence intervals to facilitate visual comparison across estimates. When comparing two estimates, non-overlapping 83% confidence intervals correspond approximately to a statistically significant difference at the 5% level [46-48]. Substantive conclusions remain unchanged when using 95% confidence intervals.”

[46] Goldstein, H., & Healy, M. J. (1995). The graphical presentation of a collection of means. *Journal of the Royal Statistical Society: Series A (Statistics in Society)*, *158*(1), 175-177.

[47] Bolsen, T., & Thornton, J. R. (2014). Overlapping confidence intervals and null hypothesis testing. *The Experimental Political Scientist*, *4*(1), 12-16.

[48] Knol, M. J., Pestman, W. R., & Grobbee, D. E. (2011). The (mis) use of overlap of confidence intervals to assess effect modification. *European journal of epidemiology*, *26*(4), 253-254.

**RESPONSES TO REVIEWER 2**

**Reviewer #2:**

*The revised version addresses all my previous comments. A few matters remain that should be addressed:*

*1. The contribution is still somewhat unclear. The paper confuses two lines of research in this sentence, p. 4: “While research has extensively documented the parental penalties faced by academic mothers, the fertility trajectories of academic men, the impact of fatherhood on their academic careers, and the ways in which career advancement influences men’s reproductive outcomes remain underexplored.” One line of research looks at the effects of children on careers. Here, it’s not true that no one looks at the effects for fathers, as often comparisons are made with the careers of mothers and fathers, also in papers on academia. The other line of research we might define looks at effects of career (tenure) on fertility. The authors need to make this distinction clear. It may be that the effects on fertility for men is less studied and the cited studies may be used to make this claim, but please clarify.*

**RESPONSE:** We thank the Reviewer for this comment. We have now clarified in the paper and the abstract the existence of two lines of research (1. Fertility affecting career progressions and 2. career progression affecting fertility), and we better situate our contribution within the second line of research. We have deleted the sentence on p.4.

*It’s clear that the authors have not used a proofreader. I found several cases where they should improve the clarity of the text. My suggestion is that they use one of the many available software solutions to go through the text to correct additional things I did not discover. Here are the ones I found:*

**RESPONSE:** We thank the Reviewer for highlighting the sentences that needed improvement. All of these sentences have been edited. The current version of the paper has now been thoroughly proofread.

*2. Abstract first sentence: “The interplay between career progression and fertility in academia remains a key—and unresolved—issue in the contemporary scholarly debate.” This is a poor sentence. In what sense is it an “issue”? Maybe the authors want to say something about the tension between career and fertility?*

**RESPONSE:** The sentence was edited as follows:

“The interplay between career progression and fertility remains a central theme in the scholarly debate on academia.”

*3. p. 1 “hence higher rates of childlessness and lower fertility for women compared to men.” “hence leading to higher rates of childlessness and lower fertility for women compared to men.”*

**RESPONSE:** Corrected.

*4. p. 2. “Policies supporting family planning for academics should be addressed alongside those targeted to reduce gender disparities in career progression.” What exactly are the authors proposing? What does “alongside those targeted” mean?*

**RESPONSE:** We have edited the sentence as follows:

“Policies aimed at reducing gender disparities in academia should address these differences in fertility by supporting academic women in transitioning to parenthood.”

*5. p. 3. Don’t understand: “The reasons for underachieving fertility goals among academics remain largely underexplored.”*

**RESPONSE:** “Underachieved fertility” refers to having fewer children than desired. We have deleted the sentence and merged the explanation with the previous sentence. The text now reads as follows (62-64 lines):

“Also, previous research has highlighted a persistent gap between the desired and actual number of children among academic women [8], and the reasons behind this gap remain largely underexplored.”

*6. p. 5. Change: “declines in their scholarly output [2, 35].” “declines in scholarly output [2, 35].”*

**RESPONSE:** Corrected.

*7. p. 7 “In the absence of a response within one week, a follow-up email was sent after one and two weeks.” “In the absence of a response within one week, a follow-up email was sent after one, and then again after two weeks.”*

**RESPONSE:** Corrected.

*8. p. 13 “indicating similar birth-spacing patterns among academic parents (SI, Tab. S3).” Something is missing here. As among non-academic parents?*

**RESPONSE:** The sentence notes that women and men in our sample tend to have the second child about 2 years after the first. We edited the text as follows (lines 299-302):

“The average interval between the first and second child is approximately two years for both men and women, indicating similar birth-spacing patterns among academic mothers and fathers.”

*9. Line 309, suggest replacing “However,” for “Also,”.*

**RESPONSE:** We have reformulated the whole sentence, also in reply to another Reviewer (lines 313-319):

“The results of Model 1 (Tab. 1) show that, after controlling for age, PhD cohort, and field of study, tenure significantly increases the hazard of becoming a parent. Among academic men, the hazard of having a child in the years following promotion increases by 62% compared to non-tenured men (OR = 1.62, p < 0.001), while among academic women it increases by 44% compared to non-tenured women (OR = 1.44, p < 0.01). The average marginal effects (AMEs) of tenure on the probability of having a first child are slightly higher for academic men than women (but the difference is not statistically significant because of overlapping CIs; see Fig. 2).”

*10. p. 14: “Also, compared with early career academics (such as PhD-seeking for a job, postdoctoral fellows),” What is PhD-seeking?*

**RESPONSE:** PhD graduates who are on the job market looking for a job. For clarity, we edited it to (line 345):

“PhD graduates seeking employment.”
