# Supplementary material for: The best time to become a parent: The relationship between tenure and fertility among academics
Source: PLoS One. 2026 Aug 12;21(8):e0353232. doi: 10.1371/journal.pone.0353232 (PMC13466013; doi:10.1371/journal.pone.0353232)
Supplement: Peer review history

# Peer review: Decision Letter 0

**DOI:** 10.1371/journal.pone.0353232.r001

**Authors:** Deni Mazrekaj

24 Oct 2025

PONE-D-25-41240

The Best Time to Become a Parent: The Effect of Tenure on Fertility Among Academics

PLOS ONE

Dear Dr. Gorodetskaya,

Thank you for submitting your manuscript to PLOS ONE. After careful consideration, we feel that it has merit but does not fully meet PLOS ONE’s publication criteria as it currently stands. Therefore, we invite you to submit a revised version of the manuscript that addresses the points raised during the review process.

Please submit your revised manuscript by Dec 08 2025 11:59PM. If you will need more time than this to complete your revisions, please reply to this message or contact the journal office at plosone@plos.org. When you're ready to submit your revision, log on to https://www.editorialmanager.com/pone/ and select the 'Submissions Needing Revision' folder to locate your manuscript file.

Please include the following items when submitting your revised manuscript:

- A rebuttal letter that responds to each point raised by the academic editor and reviewer(s). You should upload this letter as a separate file labeled 'Response to Reviewers'.
- A marked-up copy of your manuscript that highlights changes made to the original version. You should upload this as a separate file labeled 'Revised Manuscript with Track Changes'.
- An unmarked version of your revised paper without tracked changes. You should upload this as a separate file labeled 'Manuscript'.

If you would like to make changes to your financial disclosure, please include your updated statement in your cover letter. Guidelines for resubmitting your figure files are available below the reviewer comments at the end of this letter.

If applicable, we recommend that you deposit your laboratory protocols in protocols.io to enhance the reproducibility of your results. Protocols.io assigns your protocol its own identifier (DOI) so that it can be cited independently in the future. For instructions see: https://journals.plos.org/plosone/s/submission-guidelines#loc-laboratory-protocols. Additionally, PLOS ONE offers an option for publishing peer-reviewed Lab Protocol articles, which describe protocols hosted on protocols.io. Read more information on sharing protocols at https://plos.org/protocols?utm_medium=editorial-email&utm_source=authorletters&utm_campaign=protocols.

We look forward to receiving your revised manuscript.

Kind regards,

Deni Mazrekaj

Academic Editor

PLOS ONE

Journal Requirements:

When submitting your revision, we need you to address these additional requirements.

1. Please ensure that your manuscript meets PLOS ONE's style requirements, including those for file naming. The PLOS ONE style templates can be found at

https://journals.plos.org/plosone/s/file?id=wjVg/PLOSOne_formatting_sample_main_body.pdf and

https://journals.plos.org/plosone/s/file?id=ba62/PLOSOne_formatting_sample_title_authors_affiliations.pdf

2. Please also include in your MS all the information removed for blind review as peer review at PLOS One is not double-blinded (https://journals.plos.org/plosone/s/editorial-and-peer-review-process).

3. If the reviewer comments include a recommendation to cite specific previously published works, please review and evaluate these publications to determine whether they are relevant and should be cited. There is no requirement to cite these works unless the editor has indicated otherwise.

Additional Editor Comments:

The paper is very interesting and both reviewers agree that it provides a contribution to the literature. That being said, both reviewers highlight some major methodological issues, especially regarding participants' self-selection into the sample. In addition to the reviewers' comments, I would also suggest the authors to address the following:

- Clarify the precise contribution to the literature. Currently, the authors use imprecise wording such as 'largely overlooked.' Does this mean that this is the first study to investigate both men and women or not? If not, what is the precise contribution? Please avoid this kind of hedging when specifying the contribution of your study.

[Note: HTML markup is below. Please do not edit.]

Reviewers' comments:

Reviewer's Responses to Questions

**Comments to the Author**

1. Is the manuscript technically sound, and do the data support the conclusions?

The manuscript must describe a technically sound piece of scientific research with data that supports the conclusions. Experiments must have been conducted rigorously, with appropriate controls, replication, and sample sizes. The conclusions must be drawn appropriately based on the data presented.

Reviewer #1: Partly

Reviewer #2: Partly

**********

2. Has the statistical analysis been performed appropriately and rigorously?

Reviewer #1: Yes

Reviewer #2: Yes

**********

3. Have the authors made all data underlying the findings in their manuscript fully available?

The PLOS Data policy requires authors to make all data underlying the findings described in their manuscript fully available without restriction, with rare exception (please refer to the Data Availability Statement in the manuscript PDF file). The data should be provided as part of the manuscript or its supporting information, or deposited to a public repository. For example, in addition to summary statistics, the data points behind means, medians and variance measures should be available. If there are restrictions on publicly sharing data—e.g. participant privacy or use of data from a third party—those must be specified.

Reviewer #1: Yes

Reviewer #2: Yes

**********

4. Is the manuscript presented in an intelligible fashion and written in standard English?

PLOS ONE does not copyedit accepted manuscripts, so the language in submitted articles must be clear, correct, and unambiguous. Any typographical or grammatical errors should be corrected at revision, so please note any specific errors here.

Reviewer #1: Yes

Reviewer #2: No

**********

5. Review Comments to the Author

Please use the space provided to explain your answers to the questions above. You may also include additional comments for the author, including concerns about dual publication, research ethics, or publication ethics. (Please upload your review as an attachment if it exceeds 20,000 characters)

Reviewer #1: “The Best Time to Become a Parent: The Effect of Tenure on Fertility Among Academics”

Referee report

This study examines how tenure attainment is associated with transitions into first and second childbirth, as well as fertility intentions, among Italian male and female academics. The paper shows that the hazard of becoming a parent increases when both male and female academics obtain a tenured position, relative to untenured male and female academics. No significant associations are found between tenure attainment and second childbirth. Finally, among childless female academics, tenure attainment is associated with positive fertility intentions. Overall, I find the topic and paper interesting and relevant, but there are some points I would like the authors to address, as I outline below.

Main comments

- Line 155-156: Please describe in more detail in the text how your sample deviates from the population. E.g., more women, in which academic rank, in which field,…

- Line 157-156: the authors argue that “To correct for differential participation and ensure population representativeness, we applied direct post-stratification weights.” However, weighting alone cannot ensure population representativeness, as participation in the survey may still depend on unobserved factors that are correlated with your main measures of interest. For example, academics who are particularly concerned about the family–career interplay or who have salient experiences (such as having had children after tenure or not having children because of unstable positions) may be more likely to respond than those for whom these issues are less central. Such self-selection cannot be corrected through post-stratification, which adjusts only for observable characteristics. The resulting estimates should therefore be interpreted as weighted associations within the respondent sample rather than as population-level effects. The authors should acknowledge this limitation explicitly and discuss how it might influence interpretation—for instance, whether their findings could represent an upper or lower bound of the associations one would expect in the general population.

- Model 1 but also other models: is ‘younger than 35’ the most meaningful cutoff? This may already be late in the fertility timeline. Can you show the distribution of age at parenthood among female and male academics in your sample?

- Lines 206–211: How is the attainment of the National Scientific Abilitation (ASN) treated in your analysis? Following the Gelmini Reform, obtaining the ASN has become a formal prerequisite for tenure in Italy and may itself constitute a significant step toward career stability and family formation. Relatedly, it was unclear to me whether the “attainment of a tenured academic position” on line 206 referred to the actual promotion date, the date of eligibility (i.e., when the ASN was obtained), or the start date of a tenure-track position (e.g., the beginning of an RTD-B research contract). It would be interesting to replicate the analyses using ASN attainment as the key explanatory variable, to see whether this also affects fertility transitions. If the data does not allow it, it would be useful to acknowledge it in the discussion.

- I think Models 2 and 3 should be shown in the main text, either as Tables or Figures. To save space, Figure 1 could be moved to the Appendix.

- One final general comment that I have is that to compare associations between men and women, the authors should calculate average marginal effects, given that odds ratios that are estimated in different models cannot be directly compared with each other (Mood 2010). So, statements like the one in the abstract (tenure significantly increases the hazard of becoming a parent for both men and, albeit to a lesser extent, for women) or the one in the conclusion (Our findings show that men—and to a lesser extent women—tend to postpone first-time parenthood until after securing tenure), if based on results of Model 1 (and thus comparing OR=1.62 for men and OR=1.44 for women), should be backed by calculations of Average Marginal Effects.

Minor comments

- Lines 84-87: improve phrasing, it is unclear which of the themes is underexplored

- Line 110-111: specify what you mean by faculty position, who is it that you are dropping?

- Line 127: “identified through systematic keyword searches”. What keyword searches?

- Line 166: specify where this share is taken from

- Line 269: “with no notable differences between women aged below or above 35.”: no differences between women aged below 35 and between 35-39 I suppose.

- Lines 32, 301, 315, 353: why ‘young’ childless women? From Table S7 the reference category is ‘non-tenured, childless’, and the significant coefficient is for ‘Tenured, childless’, so you do not define any age category.

- I would rather always frame your findings in terms of "associations" rather than "effects" (see, e.g., line 96) given that you do not use causal methods. Talking about the ‘effect’ of tenure might be misleading.

References

Mood, C. (2010). Logistic regression: Why we cannot do what we think we can do, and what we can do about it. European sociological review, 26(1), 67-82.

Reviewer #2: This a nice paper which highlights the role of tenure and its relationship with fertility.

General comments:

I lack a clear context of the Italian case. Here are some aspects that I would like more info on:

* What do parental leave rights look like in Italy? Are they linked to tenure or do everyone have the same rights on the labor market? How might such rights influence the choice of having children?

* It would be useful to understand whether it is common for phd students to have children as it is in Nordic countries. Maybe it is not common, but it could be interesting for the reader, even though this group is not analyzed. If not common, is this because phd students are typically younger in Italy?

* I would also see some descriptive material of the data analyzed including age, position and university affiliation and how this relates to the population of academics.

Data sampling/collection:

1) A response rate of only 3.5% suggests serious risks of sample selection bias. This does not only concern selection on observables, which the authors try to address by weighting, there could also be a risk that non-observables bias the results. For instance, only those with sufficient time to answer will do so. Thus, less busy researchers may answer. Also, those particularly engaged in the topic of the survey may choose to repsond. The authors do not discuss this at all.

2) This raises issues of why not alternative data collection procedures have not been undertaken, for instance directly approaching researchers with interviews or using administrative data. The authors should discuss these objections.

Weighting:

If I understand correctly there were 84 strata by gender, but as written now there are 2 x 6 x 14 = 168 strata. Clarify this point.

Wordings:

126: "two-pronged" rather than "two-prolonged"

126-129: the distribution of the survey is not clearly described. Were 218 associations contacted and 72 distributed it? Or did you chose them? Similarly, were universities chosen by the authors for distribution, or how was this done?

p. 9-10 talks about interviews having been conducted, but it seems that a survey was distributed. My guess is that these are typos. Clarify!

215: Explain the "Gelmini reform" and how it impacted on academic career trajectories.

224: Change "those 52%" to "of which 52%"

256: "approach" is not the right word here.

**********

6. PLOS authors have the option to publish the peer review history of their article (what does this mean?). If published, this will include your full peer review and any attached files.

If you choose “no”, your identity will remain anonymous but your review may still be made public.

**Do you want your identity to be public for this peer review?**  For information about this choice, including consent withdrawal, please see our Privacy Policy.

Reviewer #1: No

Reviewer #2: No

**********

[NOTE: If reviewer comments were submitted as an attachment file, they will be attached to this email and accessible via the submission site. Please log into your account, locate the manuscript record, and check for the action link "View Attachments". If this link does not appear, there are no attachment files.]

To ensure your figures meet our technical requirements, please review our figure guidelines: https://journals.plos.org/plosone/s/figures

You may also use PLOS’s free figure tool, NAAS, to help you prepare publication quality figures: https://journals.plos.org/plosone/s/figures#loc-tools-for-figure-preparation.

NAAS will assess whether your figures meet our technical requirements by comparing each figure against our figure specifications.

Attachment Submitted filename: PlosOne review.docx

# Author comment: Author response to Decision Letter 1

**DOI:** 10.1371/journal.pone.0353232.r002

8 Dec 2025

Dear Editor,

We would like to thank you for your careful reading of our manuscript and for the very pertinent comments. We also appreciate the opportunity to revise and resubmit our work.

We have revised the text and added further descriptive and contextual information in line with the reviewers’ comments. Please find detailed responses to all reviewers’ points in the accompanying response letter.

Thank you again for dedicating your time to our work.

Best regards,

The author(s)

Responses to reviewers

Dear Reviewers,

We would like to thank you for your positive assessment of our work and for the thorough reading of our manuscript. We found your comments extremely helpful and have addressed all of them in the revised version.

Below, we provide a detailed account of how we have responded to each of your specific comments and requests. We are confident that the paper has improved thanks to your input.

Thank you again for your constructive feedback.

The author(s)

Additional Editor Comments

COMMENT: Clarify the precise contribution to the literature. Currently, the authors use imprecise wording such as 'largely overlooked.' Does this mean that this is the first study to investigate both men and women or not? If not, what is the precise contribution? Please avoid this kind of hedging when specifying the contribution of your study.

RESPONSE: Thank you for this comment. We have now clarified the contribution more precisely in the revised manuscript. To the best of our knowledge, no previous quantitative study has jointly examined fertility intentions among male and female academics using comparable measures and analytical strategies. Existing work has overwhelmingly focused on women, typically treating men as a residual category or excluding them altogether. For this reason, we now describe our contribution as filling a gap in the literature that has overwhelmingly focused on female academics, which reflects the state of the field more accurately and avoids unintended ambiguity.

RESPONSES TO REVIEWER 1

COMMENT 1: Line 155-156: Please describe in more detail in the text how your sample deviates from the population. E.g., more women, in which academic rank, in which field,…

RESPONSE: We thank the Reviewer for this suggestion. In the revised version of the manuscript, we have incorporated a detailed comparison between the sample and the target population in the Data Collection section (lines 169–176) and reported the results in a graph in the supporting information (Figure S1-S4). Please find below the text version.

A comparison between the target population and the final sample shows that their composition is broadly similar in terms of sex, academic rank, age, and disciplinary area, with only a few deviations exceeding five percentage points. Women are overrepresented in the sample by 13 percentage points (54% vs. 41%), and academics aged 30–39 by 7 percentage points. Permanent researchers are slightly underrepresented by 5 percentage points. In terms of scientific disciplines, there are no significant deviations, except for scholars in biology, who are overrepresented by 6 percentage points, and those in medicine, who are underrepresented by 7 percentage points.

COMMENT 2: Line 157-156: The authors argue that “To correct for differential participation and ensure population representativeness, we applied direct post-stratification weights.” However, weighting alone cannot ensure population representativeness, as participation in the survey may still depend on unobserved factors that are correlated with your main measures of interest. For example, academics who are particularly concerned about the family–career interplay or who have salient experiences (such as having had children after tenure or not having children because of unstable positions) may be more likely to respond than those for whom these issues are less central. Such self-selection cannot be corrected through post-stratification, which adjusts only for observable characteristics. The resulting estimates should therefore be interpreted as weighted associations within the respondent sample rather than as population-level effects. The authors should acknowledge this limitation explicitly and discuss how it might influence interpretation—for instance, whether their findings could represent an upper or lower bound of the associations one would expect in the general population.

RESPONSE: We thank the Reviewer for this comment. We agree that self-selection may be a limitation of our study. Indeed, the deviations between the population and the sample described in the previous point raised by the Reviewer support this limitation, to some extent. For this reason, in the Discussion section (lines 428-442), we explicitly addressed the possibility of self-selection in the sample and highlighted how this may represent a limitation for this type of empirical study. Please find below the text version.

We acknowledge some limitations. While we applied post-stratification weights to align our non-probability sample with the known population margins for key sociodemographic variables, thereby reducing observable discrepancies between our respondents and the target population, weighting alone cannot ensure full population representativeness, since survey participation may still depend on unobserved factors correlated with the main variables of interest [51, 52]

In our case, it is plausible that some respondents decided to participate because they were particularly concerned about the family–career interplay or had salient experiences related to it (for instance, having had children after obtaining tenure, or not having children due to job instability). Such respondents might have felt especially engaged with the topic of the survey. Conversely, those with sufficient time to complete the questionnaire might have been more likely to participate, potentially excluding researchers with children who face stronger time constraints due to competing work and family demands. Consequently, it is reasonable to assume that not only one single, homogeneous group (e.g., academics with children) is overrepresented in our sample. Despite this, whilst our weighting procedure improves the correspondence with the reference population, residual selection biases cannot be entirely ruled out [53].

COMMENT 3: Model 1 but also other models: is ‘younger than 35’ the most meaningful cutoff? This may already be late in the fertility timeline. Can you show the distribution of age at parenthood among female and male academics in your sample?

RESPONSE: We believe that this cutoff is meaningful for our sample. Since the risk set for the transition to parenthood consists of individuals who are childless at the time of PhD completion, with time at risk beginning in the year they complete their PhD, we exclude from the models all individuals who became parents before or during PhD completion (who, on average, had their first child before age 29, the mean age at PhD completion in our sample). As a result, the median age at first birth in our analytical sample is equal to 37 for men and 35 for women. The Table below reports the distribution and descriptive statistics of the age at first birth for women and men in the analytical sample.

Also, age 35 is the clinical threshold at which a first-time pregnancy is classified as advanced maternal age, with all the additional screenings and guidelines this entails (Schummers et al., 2018). This threshold is also widely used in the demographic literature to define advanced maternal age (Sobotka & Beaujouan, 2018).

Table 1. Descriptive statistics of age at first childbirth among academic mothers and fathers in the analytical sample (see "Response to Reviewer" document)

COMMENT 4: Lines 206–211: How is the attainment of the National Scientific Abilitation (ASN) treated in your analysis? Following the Gelmini Reform, obtaining the ASN has become a formal prerequisite for tenure in Italy and may itself constitute a significant step toward career stability and family formation. Relatedly, it was unclear to me whether the “attainment of a tenured academic position” on line 206 referred to the actual promotion date, the date of eligibility (i.e., when the ASN was obtained), or the start date of a tenure-track position (e.g., the beginning of an RTD-B research contract). It would be interesting to replicate the analyses using ASN attainment as the key explanatory variable, to see whether this also affects fertility transitions. If the data does not allow it, it would be useful to acknowledge it in the discussion.

RESPONSE: We thank the Reviewer for this insightful comment. We did not collect information on the year in which academics obtained their National Scientific Abilitation (ASN). The “attainment of a tenured academic position” refers to the actual year of promotion to a tenured post, not to the ASN eligibility date nor to the start of a tenure-track contract. We agree that using ASN attainment as a key explanatory variable would be interesting, but this would require both having ASN dates and restricting the analysis to younger cohorts affected by the Gelmini Reform. Following the Reviewer’s suggestion, we now mention in the discussion that future research may consider the association between attaining ASN eligibility -beyond actual promotion- and fertility.

COMMENT 5: I think Models 2 and 3 should be shown in the main text, either as Tables or Figures. To save space, Figure 1 could be moved to the Appendix.

RESPONSE: Thank you, we agree. We have included Models 2 and 3 in the main text as figures:

Figure 3. Average marginal effects (AMEs) of age-tenure group on the probability of having a first child among academic men (left) and women (right): results from Model 2 controlling for field of study and PhD cohort. Note: There are too few non-tenured men aged 45–49 who become parents in the sample; hence, the corresponding coefficient cannot be estimated, and this interaction term is omitted.

Figure 4. Average marginal effects (AMEs) of academic position on the probability of having a first child among male (left panel) and female (right panel) academics: results from Model 3 controlling for age class, field of study, and PhD cohort. Note: NTA-Prof = Non-Tenured Assistant Professor; TA-Prof = Tenured Assistant Professor; A/Full-Prof = Associate or Full Professor.

COMMENT 6: One final general comment that I have is that to compare associations between men and women, the authors should calculate average marginal effects, given that odds ratios that are estimated in different models cannot be directly compared with each other (Mood 2010). So, statements like the one in the abstract (tenure significantly increases the hazard of becoming a parent for both men and, albeit to a lesser extent, for women) or the one in the conclusion (Our findings show that men—and to a lesser extent women—tend to postpone first-time parenthood until after securing tenure), if based on results of Model 1 (and thus comparing OR=1.62 for men and OR=1.44 for women), should be backed by calculations of Average Marginal Effects.

RESPONSE: We thank the Reviewer for this insightful comment. In response, we have calculated Average Marginal Effects (AMEs) to compare the associations between men and women more accurately, as recommended. We have included these results in the main text (Figures 2-5), while maintaining the Odds Ratios (ORs) in the regression tables for consistency with the original model output. Additionally, we have edited the text to explicitly reference the significance of the comparisons based on AMEs. Please, find the description of the first model below:

The results of Model 1 (Tab.1) show that after controlling for age, PhD cohort, and field of study, tenure significantly increases the hazard of becoming a parent. For academic men, the hazard of conceiving a child in the years following promotion increases by 62% compared to non-tenured men (OR = 1.62, p < 0.001), and for academic women, by 44% compared to non-tenured women (OR = 1.44, p < 0.01). However, when comparing the average marginal effects (AMEs) of tenure on the probability of having a first child, the effect is stronger for men: the AME for academic men is 0.04, compared to 0.03 for academic women (Fig. 2).

Minor comments

- Lines 84-87: improve phrasing, it is unclear which of the themes is underexplored

RESPONSE: Thank you, we have now edited the sentence to improve clarity and, following also the Editor’s comments, we added a new sentence clarifying the contribution to previous literature (lines 84-93)

While research has extensively documented the parental penalties faced by academic mothers, the fertility trajectories of academic men, the impact of fatherhood on their academic careers, and the ways in which career advancement influences men’s reproductive outcomes remain underexplored. To date, no previous quantitative study has jointly examined and compared fertility intentions among male and female academics. Existing literature has overwhelmingly focused on women, often treating men as a residual category or excluding them altogether. However, emerging cohorts of academic fathers are experiencing increasing parenting demands as social norms shift toward greater paternal involvement in childcare. Despite this, academic men continue to have higher completed fertility rates than their female colleagues, and fatherhood is not typically associated with declines in their scholarly output

- Line 110-111: specify what you mean by faculty position, who is it that you are dropping?

RESPONSE: We have now edited the original sentence to improve clarity (lines 129-133):

The survey explicitly excluded PhD holders who had left academia before 2022. We also excluded individuals employed in non-standard academic positions within the Italian university system, including doctoral candidates, research grant holders, post-graduate fellows, collaborators on coordinated and continuous contracts, and adjunct professors. These roles fall outside the standard faculty career track and were therefore outside the target population of our study.

- Line 127: “identified through systematic keyword searches”. What keyword searches?

RESPONSE: Thank you for the comment, with which we agree. For this reason, we added in the main text the following paragraph (lines 140-152):

The search for scientific associations was carried out in Italian using keywords identifying the type of organisation (e.g., “association”, “society”), combined with the names of the 14 scientific-disciplinary areas listed in the MUR open data: “Mathematics and Computer Sciences”, “Physical Sciences”, “Chemical Sciences”, “Earth Sciences”, “Biological Sciences”, “Medical Sciences”, “Agricultural and Veterinary Sciences”, “Civil Engineering and Architecture”, “Industrial and Information Engineering”, “Ancient, Philological-Literary and Historical-Artistic Studies”, “Historical, Philosophical, Pedagogical and Psychological Sciences”, “Legal Sciences”, “Economic and Statistical Sciences”, “Political and Social Sciences”. Once the official website of a relevant scientific association was identified, we searched for contact details, typically those of the secretary or the president. In total, we identified 218 national scientific academic associations with available contact information, covering all 14 disciplinary areas. An email describing the study and requesting the dissemination of the survey was sent to these contacts. In the absence of a response within one week, a follow-up email was sent after one and two weeks. As a result, 72 scientific associations distributed the survey.

- Line 166: specify where this share is taken from

RESPONSE: We specified the source in the text (lines 181-182):

The population quotas (defined by gender × academic rank × disciplinary affiliation) were calculated using the official microdata on university personnel released by MUR.

The individual-level administrative data from the Italian Ministry of University and Research (MUR), available through the Portale dei dati dell’istruzione superiore (USTAT): https://ustat.mur.gov.it/.

Direct link: https://dati-ustat.mur.gov.it/dataset/serie-storica-sul-personale-universitario/resource/3bdba958-5c6

Attachment Submitted filename: Response to Reviewers.pdf

# Peer review: Decision Letter 1

**DOI:** 10.1371/journal.pone.0353232.r003

**Authors:** Deni Mazrekaj

10 Mar 2026

<div>PONE-D-25-41240R1

The Best Time to Become a Parent: The Relationship between Tenure and Fertility Among Academics

PLOS One

Dear Dr. Gorodetskaya,

Thank you for submitting your manuscript to PLOS ONE. After careful consideration, we feel that it has merit but does not fully meet PLOS ONE’s publication criteria as it currently stands. Therefore, we invite you to submit a revised version of the manuscript that addresses the points raised during the review process.

Please submit your revised manuscript by Apr 24 2026 11:59PM. If you will need more time than this to complete your revisions, please reply to this message or contact the journal office at plosone@plos.org. When you're ready to submit your revision, log on to https://www.editorialmanager.com/pone/ and select the 'Submissions Needing Revision' folder to locate your manuscript file.

Please include the following items when submitting your revised manuscript:

- A letter that responds to each point raised by the academic editor and reviewer(s). You should upload this letter as a separate file labeled 'Response to Reviewers'.
- A marked-up copy of your manuscript that highlights changes made to the original version. You should upload this as a separate file labeled 'Revised Manuscript with Track Changes'.
- An unmarked version of your revised paper without tracked changes. You should upload this as a separate file labeled 'Manuscript'.

-->If you would like to make changes to your financial disclosure, please include your updated statement in your cover letter. Guidelines for resubmitting your figure files are available below the reviewer comments at the end of this letter.

If applicable, we recommend that you deposit your laboratory protocols in protocols.io to enhance the reproducibility of your results. Protocols.io assigns your protocol its own identifier (DOI) so that it can be cited independently in the future. For instructions see: https://journals.plos.org/plosone/s/submission-guidelines#loc-laboratory-protocols. Additionally, PLOS ONE offers an option for publishing peer-reviewed Lab Protocol articles, which describe protocols hosted on protocols.io. Read more information on sharing protocols at https://plos.org/protocols?utm_medium=editorial-email&utm_source=authorletters&utm_campaign=protocols.

We look forward to receiving your revised manuscript.

Kind regards,

Deni Mazrekaj

Academic Editor

PLOS One

Journal Requirements:

If the reviewer comments include a recommendation to cite specific previously published works, please review and evaluate these publications to determine whether they are relevant and should be cited. There is no requirement to cite these works unless the editor has indicated otherwise.

Please review your reference list to ensure that it is complete and correct. If you have cited papers that have been retracted, please include the rationale for doing so in the manuscript text, or remove these references and replace them with relevant current references. Any changes to the reference list should be mentioned in the rebuttal letter that accompanies your revised manuscript. If you need to cite a retracted article, indicate the article’s retracted status in the References list and also include a citation and full reference for the retraction notice.

Additional Editor Comments:

Both reviewers are satisfied with the revision but still suggest some minor issues that should be fixed. I agree with them and would like to invite authors to address these remaning concerns.

[Note: HTML markup is below. Please do not edit.]

Reviewers' comments:

Reviewer's Responses to Questions

**Comments to the Author**

1. If the authors have adequately addressed your comments raised in a previous round of review and you feel that this manuscript is now acceptable for publication, you may indicate that here to bypass the “Comments to the Author” section, enter your conflict of interest statement in the “Confidential to Editor” section, and submit your "Accept" recommendation.

Reviewer #1: (No Response)

Reviewer #2: (No Response)

**********

2. Is the manuscript technically sound, and do the data support the conclusions?

The manuscript must describe a technically sound piece of scientific research with data that supports the conclusions. Experiments must have been conducted rigorously, with appropriate controls, replication, and sample sizes. The conclusions must be drawn appropriately based on the data presented.

Reviewer #1: (No Response)

Reviewer #2: Yes

**********

3. Has the statistical analysis been performed appropriately and rigorously?

Reviewer #1: (No Response)

Reviewer #2: Yes

**********

4. Have the authors made all data underlying the findings in their manuscript fully available?

The PLOS Data policy requires authors to make all data underlying the findings described in their manuscript fully available without restriction, with rare exception (please refer to the Data Availability Statement in the manuscript PDF file). The data should be provided as part of the manuscript or its supporting information, or deposited to a public repository. For example, in addition to summary statistics, the data points behind means, medians and variance measures should be available. If there are restrictions on publicly sharing data—e.g. participant privacy or use of data from a third party—those must be specified.

Reviewer #1: (No Response)

Reviewer #2: No

**********

5. Is the manuscript presented in an intelligible fashion and written in standard English?

PLOS ONE does not copyedit accepted manuscripts, so the language in submitted articles must be clear, correct, and unambiguous. Any typographical or grammatical errors should be corrected at revision, so please note any specific errors here.

Reviewer #1: (No Response)

Reviewer #2: No

**********

6. Review Comments to the Author

Please use the space provided to explain your answers to the questions above. You may also include additional comments for the author, including concerns about dual publication, research ethics, or publication ethics. (Please upload your review as an attachment if it exceeds 20,000 characters)

Reviewer #1: I am generally satisfied with revisions made by authors to the paper, I only have these remaining comments:

• “Existing literature has overwhelmingly focused on women, often treating men as a residual category or excluding them altogether. However, emerging cohorts of academic fathers are experiencing increasing parenting demands as social norms shift toward greater paternal involvement in childcare.”: Can you add references to these sentences/statements?

• Lines 309-311: is this difference between men and women statistically significant? Can you comment more on how large this difference is (also in the discussion)?

• While going over the paper again, I noticed you always have 83% confidence intervals in the figures. Why this choice? I would suggest using standard 95% confidence intervals.

Reviewer #2: The revised version addresses all my previous comments. A few matters remain that should be addressed:

1. The contribution is still somewhat unclear. The paper confuses two lines of research in this sentence p. 4: “While research has extensively documented the parental penalties faced by academic mothers, the fertility trajectories of academic men, the impact of fatherhood on their academic careers, and the ways in which career advancement influences men’s reproductive outcomes remain underexplored.” One line of research looks at the effects of children on careers. Here, it’s not true that no one looks at the effects for fathers, as often comparisons are made with the careers of mothers and fathers, also in papers on academia. The other line of research we might define looks at effects of career (tenure) on fertility. The authors need to make this distinction clear. It may be that the effects on fertility for men is less studied and the cited studies may be used to make this claim, but please clarify.

It’s clear that the authors have not used a proofreader. I found several cases where they should improve the clarity of the text. My suggestion is that they use one of the many available software solutions to go through the text to correct additional things I did not discover. Here are the ones I found:

2. Abstract first sentence: “The interplay between career progression and fertility in academia remains a key—and unresolved—issue in the contemporary scholarly debate.” This is a poor sentence. In what sense is it an “issue”? Maybe the authors want to say something about the tension between career and fertility?

3. p. 1 “hence higher rates of childlessness and lower fertility—for women compared to men.” “hence leading to higher rates of childlessness and lower fertility—for women compared to men.”

4. p. 2. “Policies supporting family planning for academics should be addressed alongside those targeted to reduce gender disparities in career progression.” What exactly are the authors proposing? What does “alongside those targeted” mean?

5. p. 3. Don’t understand: “The reasons for underachieving fertility goals among academics remain largely underexplored.”

6. p. 5. Change: “declines in their scholarly output [2, 35].” “declines in scholarly output [2, 35].”

7. p. 7 “In the absence of a response within one week, a follow-up email was sent after one and two weeks.” “In the absence of a response within one week, a follow-up email was sent after one, and then again after two weeks.”

8. p. 13 “indicating similar birth-spacing patterns among academic parents (SI, Tab. S3).” Something is missing here. As among non-academic parents?

9. Line 309, suggest replacing “However,” for “Also,”.

10. p. 14: “Also, compared with early career academics (such as PhD-seeking for a job, postdoctoral fellows),” What is PhD-seeking?

**********

7. PLOS authors have the option to publish the peer review history of their article (what does this mean?). If published, this will include your full peer review and any attached files.

If you choose “no”, your identity will remain anonymous but your review may still be made public.

**Do you want your identity to be public for this peer review?**  For information about this choice, including consent withdrawal, please see our Privacy Policy.

Reviewer #1: No

Reviewer #2: No

**********

[NOTE: If reviewer comments were submitted as an attachment file, they will be attached to this email and accessible via the submission site. Please log into your account, locate the manuscript record, and check for the action link "View Attachments". If this link does not appear, there are no attachment files.]

To ensure your figures meet our technical requirements, please review our figure guidelines: https://journals.plos.org/plosone/s/figures

You may also use PLOS’s free figure tool, NAAS, to help you prepare publication quality figures: https://journals.plos.org/plosone/s/figures#loc-tools-for-figure-preparation.

NAAS will assess whether your figures meet our technical requirements by comparing each figure against our figure specifications.

# Author comment: Author response to Decision Letter 2

**DOI:** 10.1371/journal.pone.0353232.r004

12 May 2026

Dear Editor, dear Reviewers,

We thank you for the careful and constructive evaluation of the revised manuscript.

We are grateful for the positive assessment and for your thoughtful comments and suggestions, which have helped us to refine the paper's focus further and improve its overall quality.

In addition to the revisions detailed in this response, the manuscript has been extensively revised, as suggested, to improve overall writing quality, including clarity, grammar, and flow. Please find our point-to-point responses below.

Thank you again for dedicating your time to our work.

Best regards,

The authors

RESPONSES TO REVIEWER 1

Reviewer #1:

I am generally satisfied with revisions made by authors to the paper, I only have these remaining comments:

• “Existing literature has overwhelmingly focused on women, often treating men as a residual category or excluding them altogether. However, emerging cohorts of academic fathers are experiencing increasing parenting demands as social norms shift toward greater paternal involvement in childcare.”: Can you add references to these sentences/statements?

RESPONSE: Thank you for your suggestion. We have included the following references on academic fathers in the text:

[36] - Cannito, M., & Santero, A. (2024). Balancing work, life, and care responsibilities in academia: the male perspective. Sociologia del lavoro, (2024/168). https://doi.org/10.3280/SL2024-168005

[37] - Damaske, S., Ecklund, E., Lincoln, A., & White, V. (2014). Male Scientists’ Competing Devotions to Work and Family: Changing Norms in a Male-Dominated Profession. Work and Occupations, 41, 477 - 507. https://doi.org/10.1177/0730888414539171.

The edited version of the text now reads as follows (lines 91-94):

“The existing literature has overwhelmingly focused on women, often treating men as a residual category or excluding them altogether, with only sparse, dated exceptions [8]. However, emerging cohorts of academic fathers are experiencing increasing parenting demands as social norms shift toward greater paternal involvement in childcare [36, 37, 6].

• Lines 309-311: is this difference between men and women statistically significant? Can you comment more on how large this difference is (also in the discussion)?

RESPONSE: The difference between men and women is not statistically significant, as shown by the overlapping confidence intervals of the AMEs in Figure 2. We have added a comment on this point in the Results section at lines 317-319:

“The average marginal effects (AMEs) of tenure on the probability of having a first child are slightly higher for academic men than women (but the difference is not statistically significant because of overlapping CIs; see Fig. 2).”

• While going over the paper again, I noticed you always have 83% confidence intervals in the figures. Why this choice? I would suggest using standard 95% confidence intervals.

AUTHORS' RESPONSE: We use 83% rather than 95% confidence intervals in the figures to facilitate the visual comparison of marginal effects. Previous studies (e.g., Bolsen & Thornton, 2014; Goldstein & Healy, 1995; Knol et al., 2011) demonstrated that, when comparing two estimates, nonoverlapping 83% CIs correspond approximately to a statistically significant difference at the 5% level. Using 83% intervals, therefore, allows readers to assess whether differences in predicted probabilities are statistically meaningful visually. For clarity, we have now added the following sentence in the paper (lines 293-296):

“All figures reporting average marginal effects (Figs 2-5) use 83% confidence intervals to facilitate visual comparison across estimates. When comparing two estimates, non-overlapping 83% confidence intervals correspond approximately to a statistically significant difference at the 5% level [46-48]. Substantive conclusions remain unchanged when using 95% confidence intervals.”

[46] Goldstein, H., & Healy, M. J. (1995). The graphical presentation of a collection of means. Journal of the Royal Statistical Society: Series A (Statistics in Society), 158(1), 175-177.

[47] Bolsen, T., & Thornton, J. R. (2014). Overlapping confidence intervals and null hypothesis testing. The Experimental Political Scientist, 4(1), 12-16.

[48] Knol, M. J., Pestman, W. R., & Grobbee, D. E. (2011). The (mis) use of overlap of confidence intervals to assess effect modification. European journal of epidemiology, 26(4), 253-254.

RESPONSES TO REVIEWER 2

Reviewer #2:

The revised version addresses all my previous comments. A few matters remain that should be addressed:

1. The contribution is still somewhat unclear. The paper confuses two lines of research in this sentence, p. 4: “While research has extensively documented the parental penalties faced by academic mothers, the fertility trajectories of academic men, the impact of fatherhood on their academic careers, and the ways in which career advancement influences men’s reproductive outcomes remain underexplored.” One line of research looks at the effects of children on careers. Here, it’s not true that no one looks at the effects for fathers, as often comparisons are made with the careers of mothers and fathers, also in papers on academia. The other line of research we might define looks at effects of career (tenure) on fertility. The authors need to make this distinction clear. It may be that the effects on fertility for men is less studied and the cited studies may be used to make this claim, but please clarify.

RESPONSE: We thank the Reviewer for this comment. We have now clarified in the paper and the abstract the existence of two lines of research (1. Fertility affecting career progressions and 2. career progression affecting fertility), and we better situate our contribution within the second line of research. We have deleted the sentence on p.4.

It’s clear that the authors have not used a proofreader. I found several cases where they should improve the clarity of the text. My suggestion is that they use one of the many available software solutions to go through the text to correct additional things I did not discover. Here are the ones I found:

RESPONSE: We thank the Reviewer for highlighting the sentences that needed improvement. All of these sentences have been edited. The current version of the paper has now been thoroughly proofread.

2. Abstract first sentence: “The interplay between career progression and fertility in academia remains a key—and unresolved—issue in the contemporary scholarly debate.” This is a poor sentence. In what sense is it an “issue”? Maybe the authors want to say something about the tension between career and fertility?

RESPONSE: The sentence was edited as follows:

“The interplay between career progression and fertility remains a central theme in the scholarly debate on academia.”

3. p. 1 “hence higher rates of childlessness and lower fertility for women compared to men.” “hence leading to higher rates of childlessness and lower fertility for women compared to men.”

RESPONSE: Corrected.

4. p. 2. “Policies supporting family planning for academics should be addressed alongside those targeted to reduce gender disparities in career progression.” What exactly are the authors proposing? What does “alongside those targeted” mean?

RESPONSE: We have edited the sentence as follows:

“Policies aimed at reducing gender disparities in academia should address these differences in fertility by supporting academic women in transitioning to parenthood.”

5. p. 3. Don’t understand: “The reasons for underachieving fertility goals among academics remain largely underexplored.”

RESPONSE: “Underachieved fertility” refers to having fewer children than desired. We have deleted the sentence and merged the explanation with the previous sentence. The text now reads as follows (62-64 lines):

“Also, previous research has highlighted a persistent gap between the desired and actual number of children among academic women [8], and the reasons behind this gap remain largely underexplored.”

6. p. 5. Change: “declines in their scholarly output [2, 35].” “declines in scholarly output [2, 35].”

RESPONSE: Corrected.

7. p. 7 “In the absence of a response within one week, a follow-up email was sent after one and two weeks.” “In the absence of a response within one week, a follow-up email was sent after one, and then again after two weeks.”

RESPONSE: Corrected.

8. p. 13 “indicating similar birth-spacing patterns among academic parents (SI, Tab. S3).” Something is missing here. As among non-academic parents?

RESPONSE: The sentence notes that women and men in our sample tend to have the second child about 2 years after the first. We edited the text as follows (lines 299-302):

“The average interval between the first and second child is approximately two years for both men and women, indicating similar birth-spacing patterns among academic mothers and fathers.”

9. Line 309, suggest replacing “However,” for “Also,”.

RESPONSE: We have reformulated the whole sentence, also in reply to another Reviewer (lines 313-319):

“The results of Model 1 (Tab. 1) show that, after controlling for age, PhD cohort, and field of study, tenure significantly increases the hazard of becoming a parent. Among academic men, the hazard of having a child in the years following promotion increases by 62% compared to non-tenured men (OR = 1.62, p < 0.001), while among academic women it increases by 44% compared to non-tenured women (OR = 1.44, p < 0.01). The average marginal effects (AMEs) of tenure on the probability of having a first child are slightly higher for academic men than women (but the difference is not statistically significant because of overlapping CIs; see Fig. 2).”

10. p. 14: “Also, compared with early career academics (such as PhD-seeking for a job, postdoctoral fellows),” What is PhD-seeking?

RESPONSE: PhD graduates who are on the job market looking for a job. For clarity, we edited it to (line 345):

“PhD graduates seeking employment.”

Attachment Submitted filename: Minor Revison Response_10 Aprile.docx

# Editor report: Decision Letter 2

**DOI:** 10.1371/journal.pone.0353232.r005

**Authors:** Deni Mazrekaj

21 Jun 2026

<p>The Best Time to Become a Parent: The Relationship between Tenure and Fertility Among Academics

PONE-D-25-41240R2

Dear Dr. Gorodetskaya,

We’re pleased to inform you that your manuscript has been judged scientifically suitable for publication and will be formally accepted for publication once it meets all outstanding technical requirements.

Within one week, you’ll receive an e-mail detailing the required amendments. When these have been addressed, you’ll receive a formal acceptance letter and your manuscript will be scheduled for publication.

An invoice will be generated when your article is formally accepted. Please note, if your institution has a publishing partnership with PLOS and your article meets the relevant criteria, all or part of your publication costs will be covered. Please make sure your user information is up-to-date by logging into Editorial Manager at Editorial Manager® and clicking the ‘Update My Information' link at the top of the page. For questions related to billing, please contact billing support.

If your institution or institutions have a press office, please notify them about your upcoming paper to help maximize its impact. If they’ll be preparing press materials, please inform our press team as soon as possible -- no later than 48 hours after receiving the formal acceptance. Your manuscript will remain under strict press embargo until 2 pm Eastern Time on the date of publication. For more information, please contact onepress@plos.org.

Kind regards,

Deni Mazrekaj

Academic Editor

PLOS One

Additional Editor Comments (optional):

The authors have addressed all the comments by the reviewers. The manuscript is ready for publication. I congratulate the authors on this fine and important work!

Reviewers' comments:

# Editor report: Acceptance letter

**DOI:** 10.1371/journal.pone.0353232.r006

**Authors:** Deni Mazrekaj

PONE-D-25-41240R2

PLOS One

Dear Dr. Gorodetskaya,

I'm pleased to inform you that your manuscript has been deemed suitable for publication in PLOS One. Congratulations! Your manuscript is now being handed over to our production team.

At this stage, our production department will prepare your paper for publication. This includes ensuring the following:

* All references, tables, and figures are properly cited

* All relevant supporting information is included in the manuscript submission,

* There are no issues that prevent the paper from being properly typeset

You will receive further instructions from the production team, including instructions on how to review your proof when it is ready. Please keep in mind that we are working through a large volume of accepted articles, so please give us a few days to review your paper and let you know the next and final steps.

Lastly, if your institution or institutions have a press office, please let them know about your upcoming paper now to help maximize its impact. If they'll be preparing press materials, please inform our press team within the next 48 hours. Your manuscript will remain under strict press embargo until 2 pm Eastern Time on the date of publication. For more information, please contact onepress@plos.org.

You will receive an invoice from PLOS for your publication fee after your manuscript has reached the completed accept phase. If you receive an email requesting payment before acceptance or for any other service, this may be a phishing scheme. Learn how to identify phishing emails and protect your accounts at https://explore.plos.org/phishing.

If we can help with anything else, please email us at customercare@plos.org.

Thank you for submitting your work to PLOS ONE and supporting open access.

Kind regards,

PLOS ONE Editorial Office Staff

on behalf of

Prof. dr. Deni Mazrekaj

Academic Editor

PLOS One
